# Serum iron status is associated with all-cause mortality in metabolic dysfunction-associated steatotic liver disease: a prospective, observational study

**DOI:** 10.3389/fendo.2024.1454193

**Published:** 2024-10-11

**Authors:** Ting Xia, Jie Ni, Yuqin Ni, Xinhui Wu, Kangming Du, Xuemei Wan, Xuli You

**Affiliations:** ^1^ Hospital of Chengdu University of Traditional Chinese Medicine, Chengdu, Sichuan, China; ^2^ Blood Purification Center, Hospital of Chengdu University of Traditional Chinese Medicine, Chengdu, Sichuan, China; ^3^ Department of Vascular Surgery, Hospital of Chengdu University of Traditional Chinese Medicine, Chengdu, Sichuan, China; ^4^ Department of Geriatric, Hospital of Chengdu University of Traditional Chinese Medicine, Chengdu, Sichuan, China; ^5^ Ophthalmology, Hospital of Chengdu University of Traditional Chinese Medicine, Chengdu, Sichuan, China

**Keywords:** MASLD, metabolic dysfunction-associated steatotic liver disease, iron status, mortality, prognosis, cohort study

## Abstract

**Introduction:**

Metabolic dysfunction-associated steatotic liver disease (MASLD) is the leading chronic liver disease worldwide. Emerging evidence suggests a close crosstalk between iron status and metabolic syndrome. Therefore, this cohort study aimed to investigate the relationship between serum iron status and all-cause mortality in individuals with MASLD.

**Methods:**

A total of 3393 subjects with MASLD identified by ultrasound from the Third National Health and Nutrition Examination Survey (NHANES III) were included in the analysis. Iron status indicators included serum iron, ferritin, transferrin saturation, total iron binding capacity, hemoglobin concentration, mean corpuscular hemoglobin, mean corpuscular volume, and mean corpuscular hemoglobin concentration. Cox proportional hazards models and restricted cubic spline models with adjustment for multiple confounders were applied. Stratified analyses were performed by sex and age.

**Results:**

During a median of 26.08 years of follow-up, high serum iron and transferrin saturation were significantly associated with reduced all-cause mortality in a linear pattern (*P*_overall_<0.001). Compared with the lowest quartile, individuals with serum iron and transferrin saturation in the third or fourth quartile intervals had a 20-40% reduction in long-term mortality. However, there was no independent association of serum ferritin, total iron binding capacity, and red blood cell indices with all-cause mortality in MASLD.

**Conclusion:**

This study suggests that serum iron and transferrin saturation have the potential to serve as independent biomarkers of all-cause mortality in patients with MASLD and implies the therapeutic potential of modifying iron status.

## Introduction

Non-alcoholic fatty liver disease (NAFLD) is characterized by the accumulation of fat in liver cells (1). As a highly prevalent liver disease, NAFLD places an enormous burden on health care systems and societies around the world and continues to increase at an alarming rate as the prevalence of obesity rises (2). In some regions, the prevalence of steatotic liver disease in adults is approaching 40% (3). The recent international consensus recommends the concept of metabolic dysfunction-associated steatotic liver disease (MASLD) as an alternative term to NAFLD (4, 5). As with NAFLD, MASLD is the predominant subtype of fatty liver, accounting for approximately 80% of it (3). Compared to NAFLD, the nomenclature MASLD better reflects the cause of the disease and removes the stigma associated with the disease name. Furthermore, another important difference from NAFLD is that cardiometabolic factors are emphasized in the diagnostic criteria for MASLD, so the characteristics of the MASLD population are somewhat different from those of the NAFLD population (6). Worryingly, fatty liver is strongly associated with a variety of unfavorable clinical outcomes, and recent investigations have found that significantly higher all-cause mortality is observed in the MASLD population (6). Despite considerable efforts to develop drugs to reverse liver steatosis, unfortunately, only resmetirom is currently approved for the treatment of non-alcoholic steatohepatitis (7). For the majority of patients, lifestyle interventions based on diet and exercise remain the cornerstone of disease management. Therefore, exploring new intervention targets and markers of disease progression is particularly important for the prevention and control of MASLD.

The liver is the major organ for storing iron and plays a key role in iron-related metabolism. A growing body of research suggests that hepatic iron overload may influence the severity of steatosis as well as the development of fibrosis (8–10). Excessive accumulation of iron in the liver cells may lead to further development of liver disease (11). Several population-based cross-sectional studies have revealed an association between iron status and the prevalence of fatty liver (12–15) However, evidence on whether iron status in patients with fatty liver affects future mortality is limited. Especially in the MASLD population, we know very little about this. Therefore, to explore the potential of iron status as an indicator of management or intervention in patients with MASLD, we analyzed the effect of serum iron, ferritin, transferrin saturation, total iron binding capacity (TIBC), hemoglobin concentration (HB), mean corpuscular hemoglobin (MCH), mean corpuscular volume (MCV), and mean corpuscular hemoglobin concentration (MCHC) on all-cause mortality in patients with MASLD using the third National Health and Nutrition Examination Survey (NHANES III) cohort, considering multiple levels of confounders.

## Methods

### Study population

NHANES III was a project conducted by the National Center for Health Statistics in the U.S. between 1988 and 1994. The purpose of NHANES III was to assess the health and nutritional status of adults and children on a nationwide basis (16). The multistage stratified complex sampling design allowed the survey data to be nationwide representative. Specifically, sampling was customized and weighted according to different subpopulations. Multilevel survey data were collected, including demographic, socioeconomic, laboratory tests, diet, chronic disease information, and physical examinations. Although the NHANES program is ongoing, NHANES III was selected for our study based on its availability of hepatobiliary imaging data and long-term mortality follow-up period. In the NHANES III cohort, 14,797 adults aged 20-74 years underwent liver ultrasound, and they were included for subsequent screening and analysis.

### Diagnostic criteria of MASLD

According to the diagnostic criteria proposed by the 2023 International Expert Consensus, the identification of patients with MASLD was based on hepatic ultrasound and cardiometabolic factors (5). First, all participants aged 20-74 years who were examined at a mobile screening center underwent hepatic/gallbladder ultrasound. Liver imaging data were stored as ultrasound video images and reviewed by three ultrasound readers. The readers received standardized training from radiology professionals and regular quality control was performed. Intra- and inter-rater kappa coefficients exceeded 0.6 (17). The status of hepatic steatosis was then evaluated based on the following five criteria: (1), liver to kidney contrast (2), vessel walls definition (3), parenchymal brightness (4), deep beam attenuation, and (5) gallbladder wall definition. In this study, mild to severe steatosis was defined as steatotic liver disease. Subsequently, patients with steatotic liver disease were examined for the presence of the following cardiometabolic factors (1): body mass index (BMI) >= 25 kg/m^2^ or waist circumference (WC) >=94 cm (male)/≥80 cm (female), (b) fasting glucose >=100 mg/dL, hemoglobin A1c >= 5.7%, 2-hour post load glucose levels >= 140 mg/dl, or diagnosis of type II diabetes mellitus (3), blood pressure >= 130/85 mmHg or diagnosis of hypertension (4); plasma triglycerides >= 150 mg/dL or taking lipid-lowering drugs, and (5) plasma HDL-cholesterol =<40 mg/dl (male) or =<50 mg/dl (female) (5). Finally, patients with etiologies other than cardiometabolic factors were excluded, including excessive alcohol consumption (>210 g/week for men and >140 g/week for women), HBV/HCV infection (positive serum hepatitis-B surface antigen or hepatitis-C antibody), and transferrin saturation >=50%.

### Iron status measurements

Eight indicators of iron status were included for investigation in this study: serum iron, ferritin, transferrin saturation, TIBC, HB, MCH, MCV, and MCHC. A modified automated Aii-25 colorimetric assay with an Alpkem RFA (rapid-flow analysis) system was used to determine serum iron and TIBC, and a Bio-Rad Laboratories “QuantImune Ferritin IRMA” kit was used to determine serum ferritin levels. Transferrin saturation was calculated from: serum iron/TIBC *100. A quantitative automated hematology analyzer, the Coulter Counter Model S-PLUS JR, was used to determine the hematologic parameters. Details of sample collection, storage, handling, and quality control can be found in the lab manual (https://wwwn.cdc.gov/nchs/data/nhanes3/manuals/labman.pdf).

Serum iron reflects circulating iron bound to transferrin; ferritin represents the size of iron stores in the body; TIBC is the sum of serum iron and unsaturated iron binding force; and transferrin saturation represents the ratio of serum iron to TIBC (18). Hematologic indicators were used to assess the effect of anemia status in MASLD (19). They were categorized into four groups according to quartiles to explore their potential association with all-cause mortality in patients with MASLD.

### Data on mortality

Individual participant data from NHANES III were linked to death records in the National Death Index to determine vital status. The follow-up period for each study participant was the duration of time between the NHANES III baseline examination date and date of death or the final follow-up date (December 30, 2019), whichever was earlier.

### Covariates

Multiple covariates were taken into account and adjusted to preclude potential confounding bias. Age, gender, race, marital status, household income, education, physical activity, diet quality, smoking status, body mass index, waist circumference, co-morbidities, and FIB-4 index were collected by questionnaire interview, physical examination, and/or laboratory tests. Race was categorized as Mexican-American, non-Hispanic white, non-Hispanic black, and others. The family income was measured by family income to poverty ratio and was categorized as <1, 1-5, and >5. Education level was categorized as <undergraduate and >=undergraduate. Physical activity was categorized as inactive, median, and active. The classification criteria were based on the intensity ratings of leisure activity categorizing them into moderate (MET 3-6) and vigorous (MET >6) categories. Those without recreational physical activity were defined as inactive individuals, those who performed three or more vigorous activities or five moderate-intensity activities per week were considered active, and those between the inactive and active groups were categorized as moderate individuals. Diet quality was measured based on Healthy Eating Index scores (range 0 to 100) calculated from NHANES III dietary intake data (20). Smoking status was categorized as current smoker, ex-smoker, and never smoker. Data on common chronic co-morbidities (hypertension and diabetes) and cancer history were collected. Based on diagnostic criteria, hypertension was defined as blood pressure >=90/140 mmHg, or taking anti-hypertensive drugs, or having ever been informed of a diagnosis of hypertension by a doctor; diabetes mellitus was defined as a fasting plasma glucose concentration of >=126 mg/dL, or a random plasma glucose concentration of >=200 mg/dL, or a hemoglobin A1C of >=6.5%, or an Oral Glucose Tolerance Test (OGTT) >=200 mg/dL, or taking antidiabetic medications, or having been informed of a diabetes diagnosis by a doctor. The history of cancer was determined by whether or not a cancer diagnosis had ever been made in the questionnaire interview. FIB-4 was calculated by the formula “(age (years) * AST (U/L))/((PLT [10^9^/L]) * (ALT (U/L))^1/2^)” and was used as a measure of liver fibrosis. Homeostatic Model Assessment for Insulin Resistance (HOMA-IR) was calculated by (fasting insulin in mU/L × fasting glucose in mmol/L)/22.5. Other variables, including age, BMI, C-reactive protein, and waist circumference were described and included as continuous variables for adjustment.

### Statistical analysis

We used the NHANES-recommended sample weights for statistical analyses because of the complex stratified sampling design used in NHANES III. The sample weights took into account oversampling, post-stratification, survey nonresponse, and survey design, thus making the inclusion sample nationally representative of noninstitutionalized civilians in the United States. The chi-square test and Wilcoxon rank-sum test were used to compare differences in dichotomous and continuous variables, respectively. Each of the eight indicators of iron status (serum iron, ferritin, transferrin saturation, TIBC, HB, MCH, MCV, and MCHC) was categorized into four groups based on quartiles. The Cox proportional hazards model was used to evaluate the association of all-cause mortality with different iron status levels. The population with the lowest quartile level of each iron status indicator was used as the reference group. Model 1 was a univariable model. In model 2, we adjusted for age and sex. In model 3, we additionally adjusted for race, marital status, household income, education, physical activity, diet quality, smoking status, body mass index, waist circumference, hypertension, diabetes, history of cancer, FIB-4 index, CRP, and HOMA-IR. The model 3 with full adjustment for confounders was used as the conclusive result. In addition, we applied the multivariable restricted cubic spline (RCS) model to investigate the dose-response effect of iron status indicators on mortality in the MASLD population, and the median values of each index were used as reference points.

A series of sensitivity analyses were performed to verify the robustness of the results, and reverse causality was the primary scenario considered. We excluded participants who died or were lost to follow-up within 1, 3, and 5 years, respectively, and re-performed multivariable Cox regression analyses to examine whether the conclusions changed.

All statistical analyses were two-tailed and statistical significance was set at p < 0.05. We used R version 4.3 (https://www.r-project.org/) to process and analyze the data.

## Results

**Figure 1:**
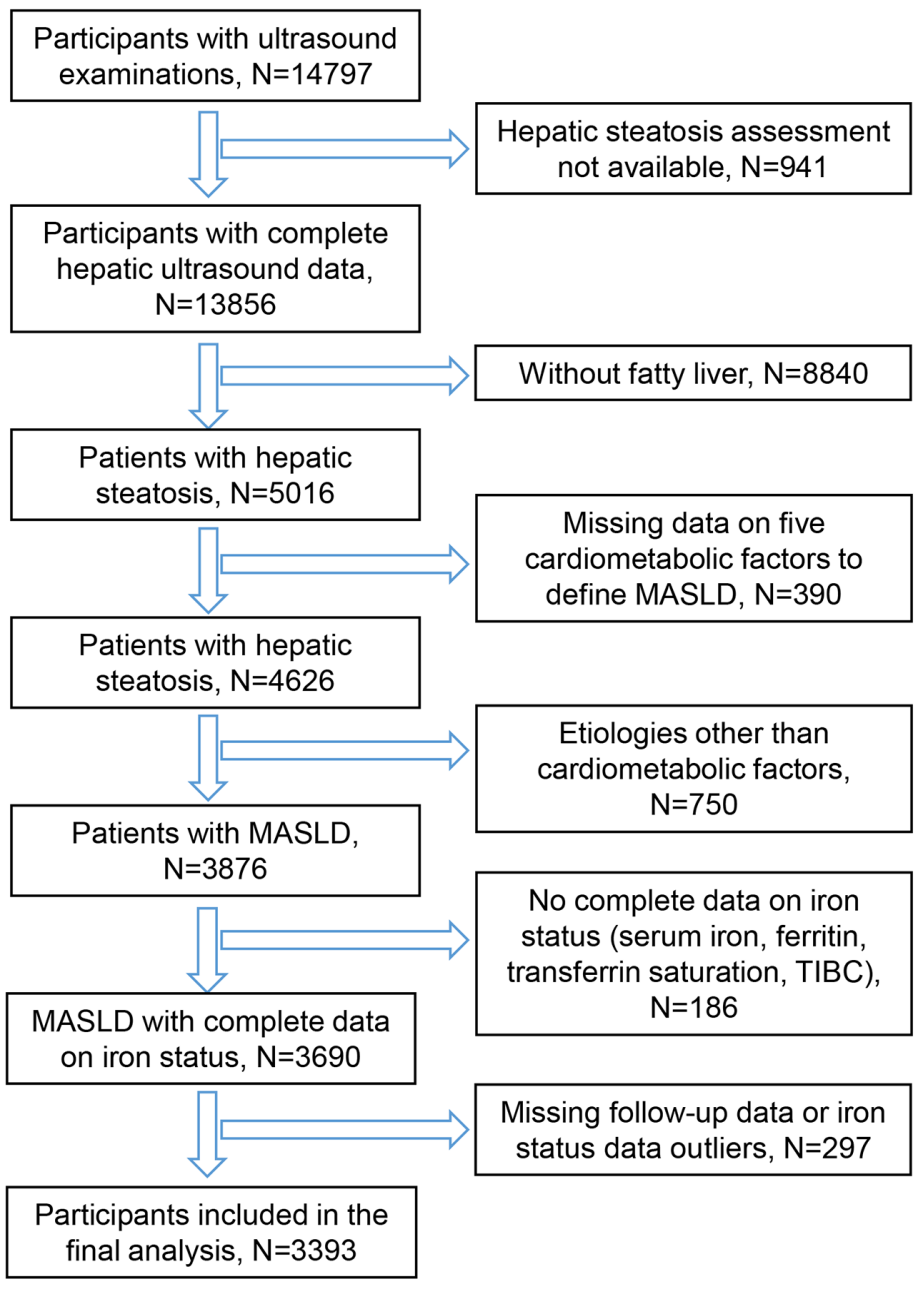
Flow chart for inclusion of participants in the analysis.

A total of 14,797 participants aged 20-74 years who underwent ultrasound were extracted from NHANES III. After screening based on eligibility criteria, 3393 participants with MASLD were finally included for analysis (**Figure 1**).

During a median 26.0 (IQR:18.3-28.0) years of follow-up, 1454 deaths occurred. The included participants were then categorized into 4 subgroups based on the quartiles of each iron status indicator. The demographic characteristics among the groups are displayed in **Supplementary Tables S1**-**S8**. It should be noted that the values of the iron status indicators were relatively more affected by age and gender. We therefore performed stratified analyses according to age and sex to examine the presence of age- and sex-specific effects.

### Serum iron and mortality of MASLD

**Figure 2:**
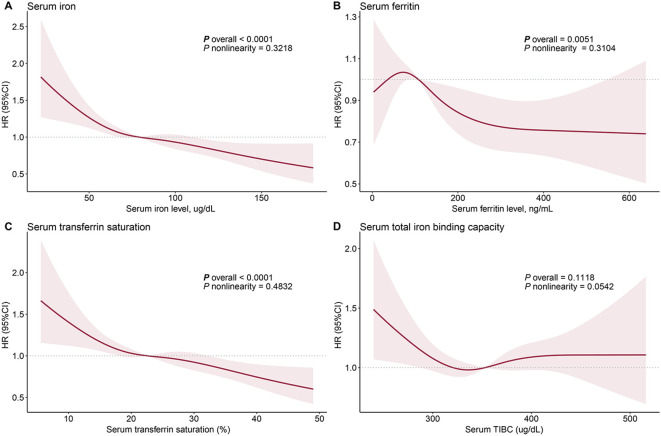
Dose-response relationship between iron status and all-cause mortality in patients with metabolic dysfunction-associated fatty liver disease: serum iron **(A)**, ferritin **(B)**, transferrin saturation **(C)**, and total iron binding capacity **(D)**. The solid red line represents the hazard ratios fitted by multivariable restricted cubic splines, and the shaded area represents the 95% confidence intervals. The median values of each iron status indicator are used as reference points. The age, gender, race, marital status, household income, education, physical activity, diet quality, smoking status, body mass index, waist circumference, hypertension, diabetes, history of cancer, FIB-4 index, C-reactive protein, and Homeostatic Model Assessment for Insulin Resistance were adjusted to control confounding factors.

We applied multiple Cox regression models to explore the association between serum iron and all-cause mortality in patients with MASLD. In the univariable model, patients with high serum iron levels tended to have a reduced risk of mortality. The association between high serum iron and low mortality was more significant after adjusting for age and sex. The multivariable model adjusting for full covariates showed that the quartile 3 group (HR= 0.73, 95%CI: 0.57-0.93, *P*=0.012) and quartile 4 group (HR=0.65, 95%CI: 0.49-0.87, *P*=0.003) were significantly associated with a lower risk of death compared with patients in the lowest quartile range (<61 ug/dL) (**Table 1**). In the multivariable RCS model, there was a significant dose-dependent effect of serum iron on mortality in MASLD (*P*_overall_ <0.001). Nonlinear associations were not observed (*P*_nonlinearity_ = 0.322). Mortality decreased significantly with increasing serum iron concentration (**Figure 2A**). To further investigate the causes of death that are potentially influenced by serum iron in patients with MASLD, we examined the correlation between serum iron levels and cancer/cardiovascular disease-specific mortality. Interestingly, we found that serum iron appeared to have an impact on cardiovascular-specific death rather than cancer-specific mortality. Patients with high level of serum iron tended to have reduced cardiovascular-specific mortality (quartile 3: HR= 0.68, 95%CI: 0.42-1.10; quartile 4: HR= 0.48, 95%CI: 0.29-0.79) (**Table 2**).

**Table 1:** Association between iron status and all-cause mortality in individuals with MASLD.

	Univariable model		Age and sex -adjusted model		Multivariable model^†^	
	HR (95% CIs)	*p-value*	HR (95% CIs)	*p-value*	HR (95% CIs)	*p-value*
Serum iron						
Quartile 1	1 [reference]		1 [reference]		1 [reference]	
Quartile 2	1.01 (0.81-1.27)	0.919	0.76 (0.59-0.97)	0.027	0.80 (0.64- 1.01)	0.065
Quartile 3	0.83 (0.68-1.02)	0.070	0.70 (0.56-0.87)	0.002	0.73 (0.57-0.93)	0.012
Quartile 4	0.70 (0.54-0.89)	0.004	0.61 (0.47-0.80)	0.001	0.65 (0.49-0.87)	0.003
Serum ferritin						
Quartile 1	1 [reference]		1 [reference]		1 [reference]	
Quartile 2	1.70 (1.35- 2.13)	<0.001	1.18 (0.88-1.59)	0.276	1.21 (0.81-1.81)	0.355
Quartile 3	1.88 (1.50-2.37)	<0.001	1.12 (0.87-1.46)	0.385	1.11 (0.81-1.52)	0.510
Quartile 4	1.74 (1.40-2.16)	<0.001	1.01 (0.77-1.32)	0.934	0.91 (0.65-1.28)	0.596
Serum transferrin saturation						
Quartile 1	1 [reference]		1 [reference]		1 [reference]	
Quartile 2	0.98 (0.77- 1.26)	0.902	0.76 (0.59-0.98)	0.035	0.83 (0.64-1.07)	0.146
Quartile 3	0.92 (0.76-1.12)	0.395	0.67 (0.54-0.85)	<0.001	0.73 (0.57-0.94)	0.015
Quartile 4	0.71 (0.56-0.90)	0.005	0.61 (0.48-0.78)	<0.001	0.67 (0.52-0.86)	0.002
Serum total iron binding capacity						
Quartile 1	1 [reference]		1 [reference]		1 [reference]	
Quartile 2	0.85 (0.68- 1.06)	0.161	0.88 (0.72-1.08)	0.231	0.84 (0.66-1.07)	0.162
Quartile 3	0.77 (0.63-0.93)	0.007	0.89 (0.71-1.13)	0.350	0.83 (0.67-1.03)	0.094
Quartile 4	0.75 (0.61-0.93)	0.007	1.07 (0.85-1.35)	0.546	0.98 (0.75-1.29)	0.890

**^†^** Adjusting for age, sex, ethnicity, marital status, smoking, education, family income level, physical activity, Healthy Eating Index, FIB-4, C-reactive protein, Homeostatic Model Assessment for Insulin Resistance, waist circumference, body mass index, hypertension, diabetes mellitus, history of malignancy.

MASLD, Metabolic dysfunction-associated steatotic liver disease; TIBC, total iron binding capacity; HR, hazard ratio; CIs, confidence intervals.

**Table 2:** Association of serum iron and transferrin saturation with cause-specific mortality in individuals with MASLD.

	Cancer-specific mortality^†^	Cardiovascular disease-specific mortality^†^
Serum iron		
Quartile 1	1 [reference]	1 [reference]
Quartile 2	0.93 (0.54- 1.58)	0.73 (0.51- 1.06)
Quartile 3	1.05 (0.65- 1.72)	0.68 (0.42- 1.10)
Quartile 4	1.12 (0.61- 2.05)	0.48 (0.29- 0.79)
Serum transferrin saturation		
Quartile 1	1 [reference]	1 [reference]
Quartile 2	1.03 (0.62- 1.70)	0.80 (0.57- 1.11)
Quartile 3	1.12 (0.67- 1.89)	0.69 (0.48- 1.00)
Quartile 4	1.07 (0.58- 1.97)	0.52 (0.33- 0.82)

MASLD, Metabolic dysfunction-associated steatotic liver disease.

**^†^** Adjusting for age, sex, ethnicity, marital status, smoking, education, family income level, physical activity, Healthy Eating Index, FIB-4, C-reactive protein, Homeostatic Model Assessment for Insulin Resistance, waist circumference, body mass index, hypertension, diabetes mellitus, history of malignancy.

### Serum ferritin and mortality of MASLD

In the univariable Cox regression analysis, high ferritin concentration was significantly associated with elevated all-cause mortality in patients with MASLD. Interestingly, this association disappeared in models adjusted for age and sex. The association between ferritin and mortality was further weakened after additional adjustment for other covariates (**Table 1**). These results suggest that the effect of ferritin on mortality is largely confounded by age and sex as well as other covariates. The multivariable RCS model found that although the overall association between ferritin and mortality was significantly associated (*P*_overall_ = 0.005), it showed a disordered effect (**Figure 2B**).

### Serum transferrin saturation and mortality of MASLD

Similar to serum iron, transferrin saturation was inversely associated with mortality in patients with MASLD. Multivariable models demonstrated a significant reduction in all-cause mortality among participants in the high-level transferrin saturation group (quartile 3: HR= 0.73, 95%CI: 0.57-0.94, *P*=0.015; quartile 4: HR= 0.67, 95%CI: 0.52-0.86, *P*=0.002) (**Table 1**). The RCS model indicated that mortality decreased with increasing serum transferrin saturation (*P*_overall _<0.001) (**Figure 2C**). Similar to serum iron, transferrin saturation appears to affect cardiovascular disease-specific but not cancer-specific mortality. High serum transferrin saturation was significantly associated with reduced cardiovascular-specific mortality (quartile 3: HR=0.69, 95%CI: 0.48-1.00; quartile 4: HR= 0.52, 95%CI: 0.33-0.82) (**Table 2**).

### Serum TIBC and mortality of MASLD

In the univariable model, serum TIBC and all-cause mortality in patients with MASLD were significantly associated; individuals with high TIBC levels had a lower risk of all-cause mortality. However, this association disappeared after adjusting for age and sex, as well as other confounders, suggesting that the association between TIBC and mortality in MASLD is confounded by age and sex (**Table 1**). The RCS model also showed no significant association between TIBC and mortality (**Figure 2D**).

### Hematologic indicators and mortality of MASLD

**Figure 3:**
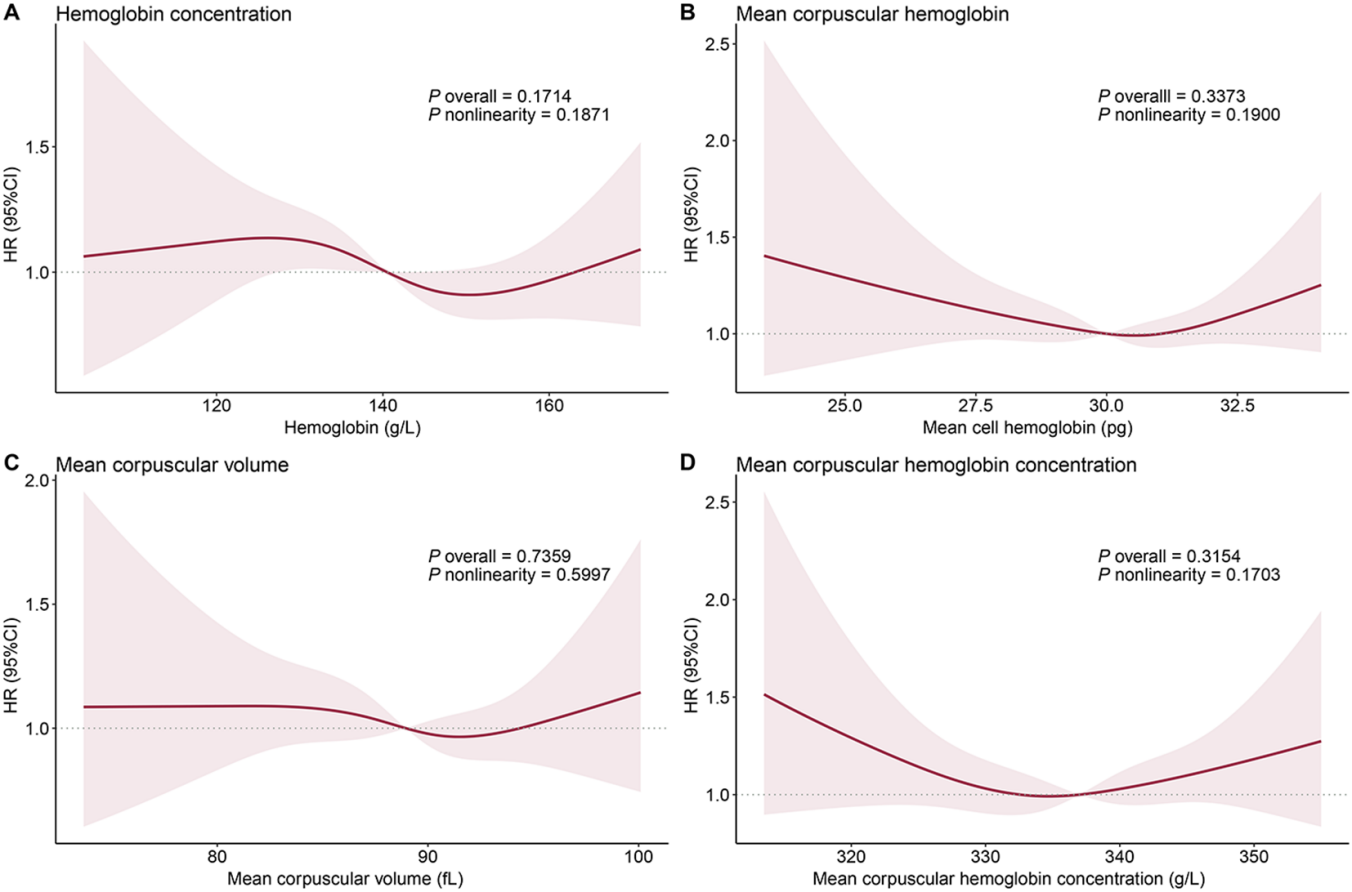
Dose-response relationship between hematologic indicators and all-cause mortality in patients with metabolic dysfunction-associated fatty liver disease: hemoglobin concentration **(A)**, mean corpuscular hemoglobin **(B)**, mean corpuscular volume **(C)**, and mean corpuscular hemoglobin concentration **(D)**. The solid red line represents the hazard ratios fitted by multivariable restricted cubic splines, and the shaded area represents the 95% confidence intervals. The median values of each iron status indicator are used as reference points. The age, gender, race, marital status, household income, education, physical activity, diet quality, smoking status, body mass index, waist circumference, hypertension, diabetes, history of cancer, FIB-4 index, C-reactive protein, and Homeostatic Model Assessment for Insulin Resistance were adjusted to control confounding factors.

Four hematologic parameters, including HB, MCH, MCV, and MCHC, were examined for correlation with all-cause mortality in patients with MASLD. The univariable analyses showed that moderate levels of HB and MCHC were significantly associated with elevated and reduced mortality, respectively (**Table 3**). However, these associations weakened after adjusting for age and sex. Following additional adjustment for other demographics factors and laboratory tests, we found no independent association between hematologic indicators and mortality in the MASLD population (**Table 3**). The RCS model confirmed the results of the Cox regression analysis (**Figure 3**).

**Table 3:** Association between hematological indicators and all-cause mortality in individuals with MASLD.

	Univariable model		Age and sex -adjusted model		Multivariable model^†^	
	HR (95% CIs)	*p-value*	HR (95% CIs)	*p-value*	HR (95% CIs)	*p-value*
Hemoglobin concentration						
Quartile 1	1 [reference]		1 [reference]		1 [reference]	
Quartile 2	1.34 (1.07-1.69)	0.012	1.06 (0.82-1.38)	0.647	0.98 (0.76- 1.28)	0.908
Quartile 3	1.28 (1.04-1.58)	0.019	1.11 (0.87-1.41)	0.391	0.90 (0.71-1.14)	0.371
Quartile 4	1.08 (0.90-1.30)	0.419	1.18 (0.92-1.53)	0.192	0.87 (0.67-1.12)	0.281
Mean corpuscular hemoglobin						
Quartile 1	1 [reference]		1 [reference]		1 [reference]	
Quartile 2	0.98 (0.76- 1.25)	0.847	0.79 (0.62-0.99)	0.043	0.81 (0.62-1.07)	0.147
Quartile 3	1.17 (0.94-1.45)	0.168	0.94 (0.711.24)	0.667	1.01 (0.77-1.33)	0.930
Quartile 4	1.21 (0.95-1.53)	0.116	0.85 (0.68-1.07)	0.158	0.87 (0.65-1.17)	0.367
Mean corpuscular volume						
Quartile 1	1 [reference]		1 [reference]		1 [reference]	
Quartile 2	1.13 (0.84- 1.52)	0.420	0.85 (0.61-1.18)	0.331	0.97 (0.58-1.63)	0.918
Quartile 3	1.13 (0.90-1.42)	0.277	0.78 (0.60-1.02)	0.065	0.93 (0.69-1.26)	0.645
Quartile 4	1.40 (1.09-1.80)	0.009	0.85 (0.64-1.11)	0.235	0.78 (0.47-1.31)	0.351
Mean corpuscular hemoglobin concentration						
Quartile 1	1 [reference]		1 [reference]		1 [reference]	
Quartile 2	0.78 (0.63- 0.96)	0.020	0.85 (0.70-1.03)	0.099	0.84 (0.56-1.26)	0.405
Quartile 3	0.81 (0.65-1.01)	0.057	0.84 (0.71-1.00)	0.048	0.87 (0.63-1.21)	0.420
Quartile 4	0.74 (0.57-0.96)	0.023	0.90 (0.72-1.14)	0.399	1.09 (0.70-1.70)	0.703

**^†^** Adjusting for age, sex, ethnicity, marital status, smoking, education, family income level, physical activity, Healthy Eating Index, FIB-4, C-reactive protein, Homeostatic Model Assessment for Insulin Resistance, waist circumference, body mass index, hypertension, diabetes mellitus, history of malignancy.

MASLD, Metabolic dysfunction-associated steatotic liver disease; TIBC, total iron binding capacity; HR, hazard ratio; CIs, confidence intervals.

### Stratified analysis

Considering the effects of age and sex on iron status in the body, we performed stratified analyses according to them. For serum iron, ferritin, transferrin saturation, and TIBC, no significant sex- and age-specific associations with mortality were observed. There was a consistent trend between groups, and differences in statistical significance in some cases may be attributed to limitations in statistical power due to sample size (**Table 4**). For hematologic indicators, there was a potential interaction effect of age on the associations of HB and MCHC with mortality, but these associations did not reach statistical significance in each of the age subgroups (**Table 5**).

**Table 4:** Stratified analysis of the association between iron status and all-cause mortality in MASLD by demographic characteristics.

	Male	Female	*p-value*_interaction_	<65 years	≥65 years	*p-value*_interaction_
Serum iron						
Quartile 1	1 [reference]	1 [reference]	*0.389*	1 [reference]	1 [reference]	*0.641*
Quartile 2	0.81 (0.53-1.24)	0.81 (0.63-1.04)		0.87 (0.65-1.16)	0.80 (0.51-1.25)	
Quartile 3	0.60 (0.38-0.94)	0.86 (0.64-1.14)		0.69 (0.48-0.99)	0.78 (0.46-1.33)	
Quartile 4	0.57 (0.36-0.90)	0.77 (0.57-1.03)		0.61 (0.44-0.83)	0.72 (0.42-1.24)	
Serum ferritin						
Quartile 1	1 [reference]	1 [reference]	*0.341*	1 [reference]	1 [reference]	*0.926*
Quartile 2	0.82 (0.51-1.34)	1.50 (0.96-2.34)		1.25 (0.82-1.92)	1.12 (0.78-1.62)	
Quartile 3	0.96 (0.64-1.52)	1.10 (0.75-1.60)		1.14 (0.82-1.57)	1.12 (0.75-1.67)	
Quartile 4	0.73 (0.44-1.24)	0.89 (0.60-1.30)		0.86 (0.60-1.24)	0.81 (0.49-1.34)	
Serum transferrin saturation						
Quartile 1	1 [reference]	1 [reference]	*0.058*	1 [reference]	1 [reference]	*0.351*
Quartile 2	0.84 (0.55-1.29)	0.78 (0.60-1.01)		0.77 (0.52-1.13)	0.76 (0.53-1.09)	
Quartile 3	0.54 (0.37-0.78)	0.97 (0.70-1.35)		0.76 (0.57-1.02)	0.63 (0.41-0.98)	
Quartile 4	0.56 (0.36-0.87)	0.73 (0.52-1.03)		0.64 (0.47-0.87)	0.73 (0.50-1.08)	
Serum total iron binding capacity						
Quartile 1	1 [reference]	1 [reference]	*0.719*	1 [reference]	1 [reference]	*0.284*
Quartile 2	0.96 (0.70-1.33)	0.73 (0.56-0.94)		0.74 (0.54-1.02)	1.06 (0.79-1.41)	
Quartile 3	0.89 (0.65-1.23)	0.72 (0.59-0.88)		0.71 (0.54-0.94)	0.79 (0.55-1.14)	
Quartile 4	1.10 (0.75-1.62)	0.85 (0.62-1.18)		0.66 (0.45-0.97)	1.12 (0.78-1.62)	

TIBC, total iron binding capacity.

**Table 5:** Stratified analysis of the association between hematological indicators and all-cause mortality in MASLD by demographic characteristics.

	Male	Female	*p-value*_interaction_	<65 years	≥65 years	*p-value*_interaction_
Hemoglobin concentration						
Quartile 1	1 [reference]	1 [reference]	*0.225*	1 [reference]	1 [reference]	*0.036*
Quartile 2	0.91 (0.55-1.51)	0.99 (0.70-1.39)		1.30 (0.86-1.95)	0.90 (0.65-1.24)	
Quartile 3	0.67 (0.42-1.09)	1.08 (0.80-1.47)		0.96 (0.64-1.42)	0.95 (0.64-1.42)	
Quartile 4	0.66 (0.43-1.03)	1.08 (0.68-1.72)		1.02 (0.65-1.59)	0.77 (0.50-1.19)	
Mean corpuscular hemoglobin						
Quartile 1	1 [reference]	1 [reference]	*0.867*	1 [reference]	1 [reference]	*0.761*
Quartile 2	0.71 (0.45-1.11)	0.87 (0.63-1.19)		0.79 (0.57-1.10)	0.90 (0.61-1.31)	
Quartile 3	1.00 (0.67-1.49)	1.02 (0.72-1.46)		0.99 (0.66-1.49)	1.04 (0.74-1.45)	
Quartile 4	0.81 (0.55-1.18)	0.94 (0.63-1.40)		0.92 (0.62-1.36)	0.93 (0.62-1.39)	
Mean corpuscular volume						
Quartile 1	1 [reference]	1 [reference]	*0.318*	1 [reference]	1 [reference]	*0.058*
Quartile 2	1.07 (0.55-2.07)	0.84 (0.43-1.66)		1.20 (0.69-2.09)	0.54 (0.30-0.98)	
Quartile 3	0.77 (0.49-1.19)	1.10 (0.62-1.94)		0.87 (0.58-1.32)	1.10 (0.68-1.78)	
Quartile 4	0.78 (0.47-1.32)	0.80 (0.39-1.67)		0.94 (0.53-1.66)	0.54 (0.29-1.01)	
Mean corpuscular hemoglobin concentration						
Quartile 1	1 [reference]	1 [reference]	*0.378*	1 [reference]	1 [reference]	*0.011*
Quartile 2	0.77 (0.43-1.37)	0.83 (0.51-1.37)		0.56 (0.31-1.03)	1.29 (0.82-2.03)	
Quartile 3	0.95 (0.62-1.45)	0.91 (0.55-1.52)		0.85 (0.51-1.40)	0.98 (0.60-1.59)	
Quartile 4	0.97 (0.55-1.70)	1.37 (0.69-2.70)		0.69 (0.36-1.32)	1.97 (1.22-3.16)	

TIBC, total iron binding capacity.

### Sensitivity analysis

To assess the robustness of the conclusions and to eliminate the effect of reverse causal associations, we excluded participants who died or were lost to follow-up within 1, 3, and 5 years, respectively, and then re-performed multivariable Cox regression analyses. Sensitivity analyses showed no change in conclusions after excluding short-term follow-up. This suggests that the results of the current study are not affected by reverse causal associations (**Table 6**).

**Table 6:** Sensitivity analyses by excluding participants who died or were lost to follow-up within 1, 3, or 5 years.

	Excluding 1 years ^a^	Excluding 3 years ^b^	Excluding 5 years ^c^
Serum iron			
Quartile 1	1 [reference]	1 [reference]	1 [reference]
Quartile 2	0.80 (0.64-1.01)	0.82 (0.65-1.03)	0.84 (0.66-1.05)
Quartile 3	0.74 (0.58-0.94)	0.75 (0.59-0.95)	0.75 (0.59-0.95)
Quartile 4	0.67 (0.50-0.89)	0.67 (0.51-0.89)	0.68 (0.52-0.89)
Serum ferritin			
Quartile 1	1 [reference]	1 [reference]	1 [reference]
Quartile 2	1.23 (0.82-1.84)	1.23 (0.82-1.84)	1.24 (0.82-1.88)
Quartile 3	1.11 (0.81-1.53)	1.11 (0.79-1.54)	1.08 (0.77-1.50)
Quartile 4	0.90 (0.64-1.27)	0.89 (0.63-1.27)	0.89 (0.63-1.27)
Serum transferrin saturation			
Quartile 1	1 [reference]	1 [reference]	1 [reference]
Quartile 2	0.83 (0.64-1.07)	0.82 (0.65-1.05)	0.82 (0.64-1.05)
Quartile 3	0.75 (0.58-0.95)	0.74 (0.58-0.96)	0.73 (0.57-0.94)
Quartile 4	0.68 (0.53-0.88)	0.68(0.53-0.88)	0.70 (0.55-0.89)
Serum total iron binding capacity			
Quartile 1	1 [reference]	1 [reference]	1 [reference]
Quartile 2	0.85 (0.67-1.08)	0.86 (0.68-1.10)	0.86 (0.67-1.11)
Quartile 3	0.82 (0.66-1.01)	0.83 (0.66-1.04)	0.79 (0.63-1.00)
Quartile 4	0.97 (0.74-1.28)	0.95 (0.71-1.26)	0.92 (0.68-1.25)
Hemoglobin concentration			
Quartile 1	1 [reference]	1 [reference]	1 [reference]
Quartile 2	0.96 (0.73- 1.27)	0.95 (0.71- 1.26)	0.92 (0.69- 1.24)
Quartile 3	0.89 (0.70-1.12)	0.86 (0.68-1.10)	0.87 (0.67-1.12)
Quartile 4	0.87 (0.67-1.13)	0.86 (0.66-1.11)	0.89 (0.68-1.17)
Mean corpuscular hemoglobin			
Quartile 1	1 [reference]	1 [reference]	1 [reference]
Quartile 2	0.81 (0.61-1.06)	0.80 (0.60-1.07)	0.79 (0.59-1.06)
Quartile 3	1.02 (0.77-1.36)	1.08 (0.81-1.44)	1.08 (0.80-1.44)
Quartile 4	0.89 (0.66-1.20)	0.93 (0.68-1.26)	0.90 (0.67-1.23)
Mean corpuscular volume			
Quartile 1	1 [reference]	1 [reference]	1 [reference]
Quartile 2	0.97 (0.58-1.63)	1.00 (0.58-1.72)	1.04 (0.60-1.80)
Quartile 3	0.92 (0.67-1.25)	0.95 (0.68-1.32)	0.95 (0.68-1.31)
Quartile 4	0.78 (0.47-1.31)	0.83 (0.50-1.39)	0.84 (0.50-1.41)
Mean corpuscular hemoglobin concentration			
Quartile 1	1 [reference]	1 [reference]	1 [reference]
Quartile 2	0.85 (0.56-1.29)	0.83 (0.56-1.22)	0.81 (0.54-1.21)
Quartile 3	0.88 (0.63-1.23)	0.88 (0.63-1.23)	0.87 (0.63-1.21)
Quartile 4	1.09 (0.69-1.71)	1.12 (0.72-1.76)	1.08 (0.69-1.68)

a Hazard ratios and corresponding confidence intervals for all-cause mortality after exclusion of MASLD patients with only one year of follow-up.

b Hazard ratios and corresponding confidence intervals for all-cause mortality after exclusion of MASLD patients with only three years of follow-up.

c Hazard ratios and corresponding confidence intervals for all-cause mortality after exclusion of MASLD patients with only five years of follow-up.

MASLD, Metabolic dysfunction-associated steatotic liver disease.

## Discussion

Iron plays an important role in various physiological processes in the human body, and imbalances in iron metabolism can lead to the development and progression of a range of diseases (21). Numerous studies have demonstrated close crosstalk and interaction of altered iron homeostasis with obesity and metabolic syndrome (e.g., glucose/lipid metabolism dysfunction, lipid peroxidation, diabetes mellitus) (22–24). MASLD, a liver disease closely associated with the metabolic syndrome, may be influenced by iron metabolism during its development. This longitudinal cohort study investigated for the first time the impact of iron status on all-cause mortality in the population with MASLD.

In a nationally representative sample of U.S. adults, our study demonstrated a dose-response association of serum iron concentration and transferrin saturation with all-cause mortality in individuals with MASLD. Multivariable models adjusting for a range of confounders indicated that individuals in the third and fourth serum iron and transferrin saturation quartiles had a 20-40% reduced risk of mortality compared with the lowest quartile group. The effect of both on mortality was mainly attributable to a reduction in cardiovascular-specific mortality. Functional iron deficiency may be related to the effect of iron status on mortality, as no association was observed between hemoglobin indicators and mortality. This study not only provides additional information for the management of individuals with MASLD, but also contributes to the understanding of the pathologic mechanisms underlying the progression of MASLD.

Before the concept of MALSD was introduced, several cross-sectional studies have investigated the association between iron status and NAFLD. Yang et al. found that high serum iron concentration was associated with lower prevalence of NAFLD (13). Zhang et al. found that serum iron, transferrin saturation, and ferritin levels were reduced in patients with NAFLD compared to the general population (15). Jung et al. found that serum ferritin levels were positively correlated with prevalence of NAFLD (14). Yu et al. found that lower serum iron and transferrin saturation levels and higher ferritin concentrations were significantly associated with increased prevalence of metabolic dysfunction-associated fatty liver disease (MAFLD) (25). Another clinical study indicated a positive correlation between serum iron levels and the presence of MAFLD in patients with type 2 diabetes (26). In summary, a large body of evidence demonstrates a significant association between iron status and the prevalence of fatty liver. In this longitudinal study, we further found that serum iron and transferrin saturation influenced future mortality in the MASLD population. Of note, a previous clinical study suggested that elevated serum ferritin was associated with increased long-term mortality in patients with NAFLD (27). Hagstrom et al. found that patients in the high and low ferritin groups had a similar risk of death until 15 years of follow-up, whereas mortality in the high ferritin group increased dramatically after more than 15 years of follow-up (27). Our study revealed a significant association between high serum ferritin levels and elevated mortality risk in the univariable model. However, this association disappeared after adjusting for age and sex. We also performed multivariable cox regression analyses in those followed for more than 15 years, and the results still suggested that the association between serum ferritin and risk of death was not statistically significant (data not shown). This discrepancy may be attributed to differences in the study samples; the current study included a MASLD population, whereas the study by Hagstrom et al. included patients with NAFLD in a clinical setting (27).

The exact mechanism underlying the effect of iron status on mortality in patients with MASLD is currently unknown. Iron status may affect mortality by directly interfering with the pathologic progression of MASLD or indirectly through association with other risk factors. In this study, the inverse association of serum iron and transferrin saturation with MASLD mortality may represent the effect of iron deficiency on MASLD. Recent studies have found that iron deficiency induces hepatocyte insulin resistance and adipogenesis through the HIF2α-ATF4 signaling pathway (8). Considering that there is no independent association between RBC-related parameters and mortality, the effect of iron status on MASLD may be related to functional iron deficiency. But more direct evidence is necessary in the future, such as the assessment of hepcidin. It is also possible that low serum iron and transferrin saturation represent infection and inflammation (28). In addition, chronic infectious diseases in childhood may also affect iron metabolism and storage by triggering inflammation and altering the development and function of the immune system, thus exerting potential long-term effects on liver health (29, 30). Therefore, whether the clinical outcomes of MASLD patients can be improved by oral iron supplementation still needs further investigation.

There are some limitations of this study that need to be noted. First, iron metabolism may change with the long-term development of MASLD. Our study only obtained iron status at the time of the baseline examination, and thus we cannot infer the impact of its dynamic changes on MASLD mortality. Second, due to the lack of medication use history, we were unable to rule out the effect of medication on participants’ iron status. In addition, we were not able to investigate specific factors affecting iron status levels, such as malnutrition, infection, inflammation, and liver damage, and further studies are needed to investigate the exact mechanisms by which iron status affects MASLD mortality. Finally, due to the nature of observational studies, the current study is still not capable of establishing a causal relationship between iron status and mortality in patients with MASLD.

## Conclusions

Our study demonstrated that iron status is closely associated with the risk of all-cause mortality in individuals with MASLD, with low serum iron and transferrin saturation independently predicting elevated mortality. Serum iron and transferrin saturation may serve as potential prognostic biomarkers for MASLD and help to better develop management strategies. Future prospective studies warrant further investigation of whether iron intake can be used as an intervention for MASLD with low serum iron.

## Data Availability

The original contributions presented in the study are included in the article/**Supplementary material**. Further inquiries can be directed to the corresponding authors.
