# Supplementary material for: Serum iron status is associated with all-cause mortality in metabolic dysfunction-associated steatotic liver disease: a prospective, observational study
Source: Front Endocrinol (Lausanne). 2024 Oct 11;15:1454193. doi: 10.3389/fendo.2024.1454193 (PMC11502310; doi:10.3389/fendo.2024.1454193)
Supplement: Supplementary file 1 [file Table1.docx]

| **Table S1.** Baseline characteristics of MASLD population by the serum iron concentration | | | | | | |
| --- | --- | --- | --- | --- | --- | --- |
| **Characteristic** | **Overall** | **Quartile 1**, <61.0 ug/dL | **Quartile 2**, 61.0-79.0 ug/dL | **Quartile 3**, 79.0-101.0 ug/dL | **Quartile 4**, >101.0 ug/dL | ***p*-value**^†^ |
| **Age (years)** | 46.90 (0.41) | 46.14 (0.65) | 48.60 (1.00) | 47.13 (0.77) | 45.86 (0.74) | 0.172 |
| **Sex** |  |  |  |  |  | <0.001 |
| Female | 1,864.0 (54.9%) | 584.0 (66.9%) | 495.0 (59.1%) | 429.0 (50.6%) | 356.0 (42.7%) |  |
| Male | 1,529.0 (45.1%) | 289.0 (33.1%) | 343.0 (40.9%) | 419.0 (49.4%) | 478.0 (57.3%) |  |
| **Ethnicity** |  |  |  |  |  | 0.035 |
| Mexican-American | 1,255.0 (37.0%) | 300.0 (34.4%) | 303.0 (36.2%) | 291.0 (34.3%) | 361.0 (43.3%) |  |
| Non-Hispanic white | 1,240.0 (36.5%) | 282.0 (32.3%) | 298.0 (35.6%) | 329.0 (38.8%) | 331.0 (39.7%) |  |
| Non-Hispanic black | 770.0 (22.7%) | 259.0 (29.7%) | 201.0 (24.0%) | 195.0 (23.0%) | 115.0 (13.8%) |  |
| Other | 128.0 (3.8%) | 32.0 (3.7%) | 36.0 (4.3%) | 33.0 (3.9%) | 27.0 (3.2%) |  |
| **Marital status** |  |  |  |  |  | 0.015 |
| Yes | 2,217.0 (65.5%) | 518.0 (59.4%) | 550.0 (65.7%) | 575.0 (68.2%) | 574.0 (69.1%) |  |
| No | 1,166.0 (34.5%) | 354.0 (40.6%) | 287.0 (34.3%) | 268.0 (31.8%) | 257.0 (30.9%) |  |
| **Family income to poverty ratio** |  |  |  |  |  | 0.011 |
| <1 | 769.0 (24.9%) | 238.0 (29.8%) | 196.0 (25.7%) | 150.0 (19.7%) | 185.0 (24.2%) |  |
| 1-5 | 2,046.0 (66.3%) | 518.0 (64.8%) | 502.0 (65.7%) | 534.0 (70.3%) | 492.0 (64.4%) |  |
| >5 | 272.0 (8.8%) | 43.0 (5.4%) | 66.0 (8.6%) | 76.0 (10.0%) | 87.0 (11.4%) |  |
| **Education** |  |  |  |  |  | 0.058 |
| <undergraduate | 2,737.0 (81.0%) | 725.0 (83.3%) | 696.0 (83.4%) | 662.0 (78.6%) | 654.0 (78.8%) |  |
| ≥undergraduate | 640.0 (19.0%) | 145.0 (16.7%) | 139.0 (16.6%) | 180.0 (21.4%) | 176.0 (21.2%) |  |
| **Physical activity** |  |  |  |  |  | 0101 |
| Inactive | 842.0 (24.8%) | 272.0 (31.2%) | 202.0 (24.1%) | 197.0 (23.2%) | 171.0 (20.5%) |  |
| Median | 1,438.0 (42.4%) | 346.0 (39.6%) | 360.0 (43.0%) | 354.0 (41.7%) | 378.0 (45.3%) |  |
| Active | 1,113.0 (32.8%) | 255.0 (29.2%) | 276.0 (32.9%) | 297.0 (35.0%) | 285.0 (34.2%) |  |
| **HEI score** | 64.15 (0.45) | 63.43 (0.60) | 64.31 (0.83) | 64.71 (0.84) | 64.12 (0.59) | 0.617 |
| **Smoking status** |  |  |  |  |  | 0.800 |
| Current smoker | 669.0 (19.7%) | 178.0 (20.4%) | 152.0 (18.1%) | 149.0 (17.6%) | 190.0 (22.8%) |  |
| Ex-smoker | 1,005.0 (29.6%) | 228.0 (26.1%) | 248.0 (29.6%) | 261.0 (30.8%) | 268.0 (32.2%) |  |
| Never smoker | 1,718.0 (50.6%) | 467.0 (53.5%) | 438.0 (52.3%) | 438.0 (51.7%) | 375.0 (45.0%) |  |
| **BMI** | 30.06 (0.28) | 31.95 (0.72) | 30.07 (0.26) | 29.76 (0.37) | 28.73 (0.29) | 0.001 |
| **WC** | 101.02 (0.66) | 103.60 (1.36) | 101.44 (0.70) | 100.73 (0.90) | 98.76 (0.78) | 0.011 |
| **Hypertension** |  |  |  |  |  | <0.001 |
| Yes | 1,487.0 (43.8%) | 418.0 (47.9%) | 401.0 (47.9%) | 379.0 (44.7%) | 289.0 (34.7%) |  |
| No | 1,906.0 (56.2%) | 455.0 (52.1%) | 437.0 (52.1%) | 469.0 (55.3%) | 545.0 (65.3%) |  |
| **Diabetes** |  |  |  |  |  | 0.051 |
| Yes | 910.0 (26.8%) | 257.0 (29.4%) | 249.0 (29.7%) | 222.0 (26.2%) | 182.0 (21.8%) |  |
| No | 2,483.0 (73.2%) | 616.0 (70.6%) | 589.0 (70.3%) | 626.0 (73.8%) | 652.0 (78.2%) |  |
| **History of Malignancy** |  |  |  |  |  | 0.167 |
| Yes | 196.0 (5.8%) | 46.0 (5.3%) | 46.0 (5.5%) | 62.0 (7.3%) | 42.0 (5.0%) |  |
| No | 3,197.0 (94.2%) | 827.0 (94.7%) | 792.0 (94.5%) | 786.0 (92.7%) | 792.0 (95.0%) |  |
| **FIB4 index** | 0.95 (0.01) | 0.88 (0.02) | 0.98 (0.03) | 0.96 (0.03) | 0.97 (0.03) | 0.008 |
| **CRP, mg/dL** | 0.47 (0.02) | 0.72 (0.05) | 0.44 (0.02) | 0.43 (0.02) | 0.32 (0.01) | <0.001 |
| **HOMA-IR** | 4.03 (0.16) | 4.60 (0.38) | 4.14 (0.26) | 3.92 (0.24) | 3.77 (0.22) | 0.028 |
| ^†^ Wilcoxon rank-sum test for complex survey samples; chi-squared test with Rao & Scott's second-order correction.  MASLD, Metabolic dysfunction-associated steatotic liver disease; HEI, Healthy eating index; BMI, body mass index; WC, waist circumference; CRP, C-reactive protein; HOMA-IR, Homeostatic Model Assessment for Insulin Resistance  Data are presented as mean (standard error) or number (percentage). | | | | | | |

| **Table S2.** Baseline characteristics of MASLD population by the serum ferritin concentration | | | | | | |
| --- | --- | --- | --- | --- | --- | --- |
| **Characteristic** | **Overall** | **Quartile 1**, <51.0 ng/mL | **Quartile 2**, 51.0-108.0 ng/mL | **Quartile 3**, 108.0-199.0 ng/mL | **Quartile 4**, >199.0 ng/mL | ***p*-value**^†^ |
| **Age (years)** | 46.90 (0.41) | 42.64 (0.57) | 46.51 (0.65) | 48.72 (0.69) | 49.27 (0.78) | <0.001 |
| **Sex** |  |  |  |  |  | <0.001 |
| Female | 1,864.0 (54.9%) | 737.0 (85.8%) | 508.0 (59.7%) | 363.0 (43.4%) | 256.0 (30.2%) |  |
| Male | 1,529.0 (45.1%) | 122.0 (14.2%) | 343.0 (40.3%) | 473.0 (56.6%) | 591.0 (69.8%) |  |
| **Ethnicity** |  |  |  |  |  | 0.012 |
| Mexican-American | 1,255.0 (37.0%) | 366.0 (42.6%) | 311.0 (36.5%) | 321.0 (38.4%) | 257.0 (30.3%) |  |
| Non-Hispanic white | 1,240.0 (36.5%) | 269.0 (31.3%) | 333.0 (39.1%) | 306.0 (36.6%) | 332.0 (39.2%) |  |
| Non-Hispanic black | 770.0 (22.7%) | 183.0 (21.3%) | 168.0 (19.7%) | 190.0 (22.7%) | 229.0 (27.0%) |  |
| Other | 128.0 (3.8%) | 41.0 (4.8%) | 39.0 (4.6%) | 19.0 (2.3%) | 29.0 (3.4%) |  |
| **Marital status** |  |  |  |  |  | 0.048 |
| Yes | 2,217.0 (65.5%) | 526.0 (61.4%) | 543.0 (64.1%) | 568.0 (68.0%) | 580.0 (68.6%) |  |
| No | 1,166.0 (34.5%) | 330.0 (38.6%) | 304.0 (35.9%) | 267.0 (32.0%) | 265.0 (31.4%) |  |
| **Family income to poverty ratio** |  |  |  |  |  | 0.010 |
| <1 | 769.0 (24.9%) | 243.0 (30.4%) | 199.0 (26.1%) | 156.0 (20.7%) | 171.0 (22.2%) |  |
| 1-5 | 2,046.0 (66.3%) | 507.0 (63.5%) | 498.0 (65.4%) | 527.0 (69.8%) | 514.0 (66.7%) |  |
| >5 | 272.0 (8.8%) | 49.0 (6.1%) | 65.0 (8.5%) | 72.0 (9.5%) | 86.0 (11.2%) |  |
| **Education** |  |  |  |  |  | 0.805 |
| <undergraduate | 2,737.0 (81.0%) | 701.0 (82.1%) | 677.0 (80.1%) | 675.0 (80.7%) | 684.0 (81.2%) |  |
| ≥undergraduate | 640.0 (19.0%) | 153.0 (17.9%) | 168.0 (19.9%) | 161.0 (19.3%) | 158.0 (18.8%) |  |
| **Physical activity** |  |  |  |  |  | 0.182 |
| Inactive | 842.0 (24.8%) | 262.0 (30.5%) | 195.0 (22.9%) | 198.0 (23.7%) | 187.0 (22.1%) |  |
| Median | 1,438.0 (42.4%) | 351.0 (40.9%) | 380.0 (44.7%) | 351.0 (42.0%) | 356.0 (42.0%) |  |
| Active | 1,113.0 (32.8%) | 246.0 (28.6%) | 276.0 (32.4%) | 287.0 (34.3%) | 304.0 (35.9%) |  |
| **HEI score** | 64.15 (0.45) | 63.30 (0.78) | 65.68 (0.53) | 63.34 (1.00) | 64.17 (0.85) | 0.055 |
| **Smoking status** |  |  |  |  |  | 0.011 |
| Current smoker | 669.0 (19.7%) | 146.0 (17.0%) | 181.0 (21.3%) | 173.0 (20.7%) | 169.0 (20.0%) |  |
| Ex-smoker | 1,005.0 (29.6%) | 180.0 (21.0%) | 230.0 (27.1%) | 274.0 (32.8%) | 321.0 (37.9%) |  |
| Never smoker | 1,718.0 (50.6%) | 533.0 (62.0%) | 439.0 (51.6%) | 389.0 (46.5%) | 357.0 (42.1%) |  |
| **BMI** | 30.06 (0.28) | 29.26 (0.59) | 30.06 (0.47) | 29.77 (0.34) | 31.01 (0.38) | 0.002 |
| **WC** | 101.02 (0.66) | 95.84 (1.23) | 100.16 (1.06) | 101.40 (0.84) | 105.95 (0.91) | <0.001 |
| **Hypertension** |  |  |  |  |  | 0.002 |
| Yes | 1,487.0 (43.8%) | 277.0 (32.2%) | 363.0 (42.7%) | 405.0 (48.4%) | 442.0 (52.2%) |  |
| No | 1,906.0 (56.2%) | 582.0 (67.8%) | 488.0 (57.3%) | 431.0 (51.6%) | 405.0 (47.8%) |  |
| **Diabetes** |  |  |  |  |  | <0.001 |
| Yes | 910.0 (26.8%) | 148.0 (17.2%) | 193.0 (22.7%) | 238.0 (28.5%) | 331.0 (39.1%) |  |
| No | 2,483.0 (73.2%) | 711.0 (82.8%) | 658.0 (77.3%) | 598.0 (71.5%) | 516.0 (60.9%) |  |
| **Malignancy** |  |  |  |  |  | 0.546 |
| Yes | 196.0 (5.8%) | 44.0 (5.1%) | 41.0 (4.8%) | 62.0 (7.4%) | 49.0 (5.8%) |  |
| No | 3,197.0 (94.2%) | 815.0 (94.9%) | 810.0 (95.2%) | 774.0 (92.6%) | 798.0 (94.2%) |  |
| **FIB4 index** | 0.95 (0.01) | 0.83 (0.02) | 0.90 (0.02) | 1.01 (0.02) | 1.03 (0.03) | <0.001 |
| **CRP, mg/dL** | 0.47 (0.02) | 0.50 (0.03) | 0.48 (0.03) | 0.42 (0.02) | 0.47 (0.03) | 0.385 |
| **HOMA-IR** | 4.03 (0.16) | 3.49 (0.26) | 3.53 (0.18) | 3.64 (0.24) | 5.25 (0.33) | <0.001 |
| ^†^ Wilcoxon rank-sum test for complex survey samples; chi-squared test with Rao & Scott's second-order correction.  MASLD, Metabolic dysfunction-associated steatotic liver disease; HEI, Healthy eating index; BMI, body mass index; WC, waist circumference; CRP, C-reactive protein; HOMA-IR, Homeostatic Model Assessment for Insulin Resistance  Data are presented as mean (standard error) or number (percentage). | | | | | | |
|  | | | | | | |

| **Table S3.** Baseline characteristics of MASLD population by the serum transferrin saturation | | | | | | |
| --- | --- | --- | --- | --- | --- | --- |
| **Characteristic** | **Overall** | **Quartile 1**, <17.2% | **Quartile 2**, 17.2-22.6% | **Quartile 3**, 22.6-29.1% | **Quartile 4**, >29.1% | ***p*-value**^†^ |
| **Age (years)** | 46.90 (0.41) | 45.50 (0.79) | 48.25 (0.97) | 48.31 (0.96) | 45.55 (0.91) | 0.071 |
| **Sex** |  |  |  |  |  | <0.001 |
| Female | 1,864.0 (54.9%) | 609.0 (71.6%) | 488.0 (57.5%) | 422.0 (49.7%) | 345.0 (40.8%) |  |
| Male | 1,529.0 (45.1%) | 242.0 (28.4%) | 360.0 (42.5%) | 427.0 (50.3%) | 500.0 (59.2%) |  |
| **Ethnicity** |  |  |  |  |  | 0.140 |
| Mexican-American | 1,255.0 (37.0%) | 318.0 (37.4%) | 297.0 (35.0%) | 305.0 (35.9%) | 335.0 (39.6%) |  |
| Non-Hispanic white | 1,240.0 (36.5%) | 273.0 (32.1%) | 307.0 (36.2%) | 324.0 (38.2%) | 336.0 (39.8%) |  |
| Non-Hispanic black | 770.0 (22.7%) | 230.0 (27.0%) | 208.0 (24.5%) | 185.0 (21.8%) | 147.0 (17.4%) |  |
| Other | 128.0 (3.8%) | 30.0 (3.5%) | 36.0 (4.2%) | 35.0 (4.1%) | 27.0 (3.2%) |  |
| **Marry** |  |  |  |  |  | 0.004 |
| married | 2,217.0 (65.5%) | 507.0 (59.6%) | 549.0 (64.9%) | 580.0 (68.5%) | 581.0 (69.2%) |  |
| unmarried | 1,166.0 (34.5%) | 343.0 (40.4%) | 297.0 (35.1%) | 267.0 (31.5%) | 259.0 (30.8%) |  |
| **Family income to poverty ratio** |  |  |  |  |  | 0.001 |
| <1 | 769.0 (24.9%) | 247.0 (31.7%) | 187.0 (24.1%) | 154.0 (20.1%) | 181.0 (23.6%) |  |
| 1-5 | 2,046.0 (66.3%) | 490.0 (62.9%) | 522.0 (67.3%) | 529.0 (69.1%) | 505.0 (65.9%) |  |
| >5 | 272.0 (8.8%) | 42.0 (5.4%) | 67.0 (8.6%) | 83.0 (10.8%) | 80.0 (10.4%) |  |
| **Education** |  |  |  |  |  | 0.048 |
| <undergraduate | 2,737.0 (81.0%) | 703.0 (82.9%) | 696.0 (82.4%) | 677.0 (79.9%) | 661.0 (79.0%) |  |
| ≥undergraduate | 640.0 (19.0%) | 145.0 (17.1%) | 149.0 (17.6%) | 170.0 (20.1%) | 176.0 (21.0%) |  |
| **Physical activity** |  |  |  |  |  | 0.110 |
| Inactive | 842.0 (24.8%) | 264.0 (31.0%) | 207.0 (24.4%) | 190.0 (22.4%) | 181.0 (21.4%) |  |
| Median | 1,438.0 (42.4%) | 347.0 (40.8%) | 351.0 (41.4%) | 361.0 (42.5%) | 379.0 (44.9%) |  |
| Active | 1,113.0 (32.8%) | 240.0 (28.2%) | 290.0 (34.2%) | 298.0 (35.1%) | 285.0 (33.7%) |  |
| **HEI score** | 64.15 (0.45) | 63.55 (0.68) | 63.67 (0.85) | 65.49 (0.82) | 63.84 (0.56) | 0.095 |
| **Smoking status** |  |  |  |  |  | 0.497 |
| Current smoker | 669.0 (19.7%) | 168.0 (19.7%) | 150.0 (17.7%) | 147.0 (17.3%) | 204.0 (24.2%) |  |
| Ex-smoker | 1,005.0 (29.6%) | 212.0 (24.9%) | 248.0 (29.2%) | 270.0 (31.8%) | 275.0 (32.6%) |  |
| Never smoker | 1,718.0 (50.6%) | 471.0 (55.3%) | 450.0 (53.1%) | 432.0 (50.9%) | 365.0 (43.2%) |  |
| **BMI** | 30.06 (0.28) | 32.01 (0.70) | 30.31 (0.37) | 29.79 (0.34) | 28.53 (0.30) | <0.001 |
| **WC** | 101.02 (0.66) | 103.42 (1.33) | 101.61 (0.78) | 101.51 (0.78) | 98.11 (0.83) | 0.001 |
| **Hypertension** |  |  |  |  |  | 0.000 |
| Yes | 1,487.0 (43.8%) | 391.0 (45.9%) | 416.0 (49.1%) | 371.0 (43.7%) | 309.0 (36.6%) |  |
| No | 1,906.0 (56.2%) | 460.0 (54.1%) | 432.0 (50.9%) | 478.0 (56.3%) | 536.0 (63.4%) |  |
| **Diabetes** |  |  |  |  |  | 0.022 |
| Yes | 910.0 (26.8%) | 235.0 (27.6%) | 248.0 (29.2%) | 237.0 (27.9%) | 190.0 (22.5%) |  |
| No | 2,483.0 (73.2%) | 616.0 (72.4%) | 600.0 (70.8%) | 612.0 (72.1%) | 655.0 (77.5%) |  |
| **Malignancy** |  |  |  |  |  | 0.936 |
| Yes | 196.0 (5.8%) | 47.0 (5.5%) | 48.0 (5.7%) | 50.0 (5.9%) | 51.0 (6.0%) |  |
| No | 3,197.0 (94.2%) | 804.0 (94.5%) | 800.0 (94.3%) | 799.0 (94.1%) | 794.0 (94.0%) |  |
| **FIB4 index** | 0.95 (0.01) | 0.85 (0.02) | 0.98 (0.04) | 1.01 (0.03) | 0.94 (0.03) | <0.001 |
| **CRP, mg/dL** | 0.47 (0.02) | 0.71 (0.05) | 0.47 (0.03) | 0.40 (0.02) | 0.33 (0.02) | <0.001 |
| **HOMA-IR** | 4.03 (0.16) | 4.86 (0.41) | 3.94 (0.33) | 4.16 (0.28) | 3.58 (0.26) | 0.010 |
| ^†^ Wilcoxon rank-sum test for complex survey samples; chi-squared test with Rao & Scott's second-order correction.  MASLD, Metabolic dysfunction-associated steatotic liver disease; HEI, Healthy eating index; BMI, body mass index; WC, waist circumference; CRP, C-reactive protein; HOMA-IR, Homeostatic Model Assessment for Insulin Resistance  Data are presented as mean (standard error) or number (percentage). | | | | | | |

| **Table S4.** Baseline characteristics of MASLD population by the serum TIBC | | | | | | |
| --- | --- | --- | --- | --- | --- | --- |
| **Characteristic** | **Overall** | **Quartile 1**, <319.0 ug/dL | **Quartile 2**, 319.0-353.0 ug/dL | **Quartile 3**, 353.0-390.0 ug/dL | **Quartile 4**, >390.0 ug/dL | ***p*-value**^†^ |
| **Age (years)** | 46.90 (0.41) | 48.68 (0.76) | 48.11 (0.88) | 46.23 (0.71) | 44.58 (0.67) | 0.004 |
| **Sex** |  |  |  |  |  | 0.001 |
| Female | 1,864.0 (54.9%) | 428.0 (50.1%) | 435.0 (50.2%) | 441.0 (53.1%) | 560.0 (66.5%) |  |
| Male | 1,529.0 (45.1%) | 426.0 (49.9%) | 432.0 (49.8%) | 389.0 (46.9%) | 282.0 (33.5%) |  |
| **Ethnicity** |  |  |  |  |  | 0.055 |
| Mexican-American | 1,255.0 (37.0%) | 275.0 (32.2%) | 288.0 (33.2%) | 322.0 (38.8%) | 370.0 (43.9%) |  |
| Non-Hispanic white | 1,240.0 (36.5%) | 301.0 (35.2%) | 331.0 (38.2%) | 324.0 (39.0%) | 284.0 (33.7%) |  |
| Non-Hispanic black | 770.0 (22.7%) | 250.0 (29.3%) | 217.0 (25.0%) | 157.0 (18.9%) | 146.0 (17.3%) |  |
| Other | 128.0 (3.8%) | 28.0 (3.3%) | 31.0 (3.6%) | 27.0 (3.3%) | 42.0 (5.0%) |  |
| **Marry** |  |  |  |  |  | 0.062 |
| Yes | 2,217.0 (65.5%) | 553.0 (65.1%) | 571.0 (66.1%) | 561.0 (67.7%) | 532.0 (63.3%) |  |
| No | 1,166.0 (34.5%) | 297.0 (34.9%) | 293.0 (33.9%) | 268.0 (32.3%) | 308.0 (36.7%) |  |
| **PIR** |  |  |  |  |  | 0.340 |
| <1 | 769.0 (24.9%) | 181.0 (23.5%) | 196.0 (24.6%) | 166.0 (22.3%) | 226.0 (29.2%) |  |
| 1-5 | 2,046.0 (66.3%) | 530.0 (68.8%) | 540.0 (67.8%) | 495.0 (66.4%) | 481.0 (62.1%) |  |
| >5 | 272.0 (8.8%) | 59.0 (7.7%) | 60.0 (7.5%) | 85.0 (11.4%) | 68.0 (8.8%) |  |
| **Education** |  |  |  |  |  | 0.585 |
| <undergraduate | 2,737.0 (81.0%) | 698.0 (82.2%) | 695.0 (80.6%) | 660.0 (79.8%) | 684.0 (81.5%) |  |
| ≥undergraduate | 640.0 (19.0%) | 151.0 (17.8%) | 167.0 (19.4%) | 167.0 (20.2%) | 155.0 (18.5%) |  |
| **Physical activity** |  |  |  |  |  | 0.246 |
| Inactive | 842.0 (24.8%) | 223.0 (26.1%) | 195.0 (22.5%) | 191.0 (23.0%) | 233.0 (27.7%) |  |
| Median | 1,438.0 (42.4%) | 353.0 (41.3%) | 372.0 (42.9%) | 388.0 (46.7%) | 325.0 (38.6%) |  |
| Active | 1,113.0 (32.8%) | 278.0 (32.6%) | 300.0 (34.6%) | 251.0 (30.2%) | 284.0 (33.7%) |  |
| **HEI score** | 64.15 (0.45) | 62.83 (0.75) | 64.18 (0.76) | 65.09 (0.78) | 64.38 (0.85) | 0.099 |
| **Smoking status** |  |  |  |  |  | 0.903 |
| Current smoker | 669.0 (19.7%) | 171.0 (20.0%) | 186.0 (21.5%) | 167.0 (20.1%) | 145.0 (17.2%) |  |
| Ex-smoker | 1,005.0 (29.6%) | 263.0 (30.8%) | 257.0 (29.6%) | 257.0 (31.0%) | 228.0 (27.1%) |  |
| Never smoker | 1,718.0 (50.6%) | 420.0 (49.2%) | 424.0 (48.9%) | 405.0 (48.9%) | 469.0 (55.7%) |  |
| **BMI** | 30.06 (0.28) | 29.44 (0.34) | 29.80 (0.36) | 30.11 (0.33) | 30.91 (0.53) | 0.134 |
| **WC** | 101.02 (0.66) | 99.42 (0.86) | 101.24 (0.96) | 101.84 (0.86) | 101.43 (1.08) | 0.045 |
| **Hypertension** |  |  |  |  |  | 0.584 |
| Yes | 1,487.0 (43.8%) | 409.0 (47.9%) | 378.0 (43.6%) | 358.0 (43.1%) | 342.0 (40.6%) |  |
| No | 1,906.0 (56.2%) | 445.0 (52.1%) | 489.0 (56.4%) | 472.0 (56.9%) | 500.0 (59.4%) |  |
| **Diabetes** |  |  |  |  |  | 0.608 |
| Yes | 910.0 (26.8%) | 255.0 (29.9%) | 226.0 (26.1%) | 221.0 (26.6%) | 208.0 (24.7%) |  |
| No | 2,483.0 (73.2%) | 599.0 (70.1%) | 641.0 (73.9%) | 609.0 (73.4%) | 634.0 (75.3%) |  |
| **History of Malignancy** |  |  |  |  |  | 0.852 |
| Yes | 196.0 (5.8%) | 50.0 (5.9%) | 53.0 (6.1%) | 46.0 (5.5%) | 47.0 (5.6%) |  |
| No | 3,197.0 (94.2%) | 804.0 (94.1%) | 814.0 (93.9%) | 784.0 (94.5%) | 795.0 (94.4%) |  |
| **FIB4 index** | 0.95 (0.01) | 1.00 (0.03) | 0.98 (0.03) | 0.90 (0.02) | 0.92 (0.03) | 0.001 |
| **CRP, mg/dL** | 0.47 (0.02) | 0.46 (0.03) | 0.45 (0.03) | 0.46 (0.02) | 0.51 (0.03) | 0.165 |
| **HOMA-IR** | 4.03 (0.16) | 3.33 (0.19) | 3.76 (0.23) | 3.83 (0.17) | 5.35 (0.41) | <0.001 |
| ^†^ Wilcoxon rank-sum test for complex survey samples; chi-squared test with Rao & Scott's second-order correction.  TIBC, Total iron binding capacity; MASLD, Metabolic dysfunction-associated steatotic liver disease; HEI, Healthy eating index; BMI, body mass index; WC, waist circumference; CRP, C-reactive protein; HOMA-IR, Homeostatic Model Assessment for Insulin Resistance  Data are presented as mean (standard error) or number (percentage). | | | | | | |

| **Table S5.** Baseline characteristics of MASLD population by the hemoglobin concentration | | | | | | |
| --- | --- | --- | --- | --- | --- | --- |
| **Characteristic** | **Overall** | **Quartile 1**, <131.0 g/L | **Quartile 2**, 131.0-140.5 g/L | **Quartile 3**, 140.5-150.5 g/L | **Quartile 4**, >150.5 g/L | ***p*-value**^†^ |
| **Age (years)** | 46.90 (0.41) | 46.07 (0.76) | 48.51 (0.76) | 48.54 (0.67) | 44.68 (0.74) | 0.001 |
| **Sex** |  |  |  |  |  | <0.001 |
| Female | 1,864.0 (54.9%) | 767.0 (90.3%) | 666.0 (78.4%) | 345.0 (40.4%) | 86.0 (10.2%) |  |
| Male | 1,529.0 (45.1%) | 82.0 (9.7%) | 184.0 (21.6%) | 508.0 (59.6%) | 755.0 (89.8%) |  |
| **Ethnicity** |  |  |  |  |  | <0.001 |
| Mexican-American | 1,255.0 (37.0%) | 266.0 (31.3%) | 285.0 (33.5%) | 329.0 (38.6%) | 375.0 (44.6%) |  |
| Non-Hispanic white | 1,240.0 (36.5%) | 210.0 (24.7%) | 329.0 (38.7%) | 342.0 (40.1%) | 359.0 (42.7%) |  |
| Non-Hispanic black | 770.0 (22.7%) | 336.0 (39.6%) | 194.0 (22.8%) | 160.0 (18.8%) | 80.0 (9.5%) |  |
| Other | 128.0 (3.8%) | 37.0 (4.4%) | 42.0 (4.9%) | 22.0 (2.6%) | 27.0 (3.2%) |  |
| **Marry** |  |  |  |  |  | <0.001 |
| Yes | 2,217.0 (65.5%) | 460.0 (54.4%) | 528.0 (62.3%) | 595.0 (69.9%) | 634.0 (75.6%) |  |
| No | 1,166.0 (34.5%) | 385.0 (45.6%) | 320.0 (37.7%) | 256.0 (30.1%) | 205.0 (24.4%) |  |
| **PIR** |  |  |  |  |  | <0.001 |
| <1 | 769.0 (24.9%) | 248.0 (31.7%) | 191.0 (24.9%) | 164.0 (21.3%) | 166.0 (21.6%) |  |
| 1-5 | 2,046.0 (66.3%) | 488.0 (62.4%) | 519.0 (67.8%) | 533.0 (69.1%) | 506.0 (65.9%) |  |
| >5 | 272.0 (8.8%) | 46.0 (5.9%) | 56.0 (7.3%) | 74.0 (9.6%) | 96.0 (12.5%) |  |
| **Education** |  |  |  |  |  | 0.203 |
| <undergraduate | 2,737.0 (81.0%) | 700.0 (83.0%) | 690.0 (81.4%) | 689.0 (81.2%) | 658.0 (78.6%) |  |
| ≥undergraduate | 640.0 (19.0%) | 143.0 (17.0%) | 158.0 (18.6%) | 160.0 (18.8%) | 179.0 (21.4%) |  |
| **Physical activity** |  |  |  |  |  | 0.040 |
| Inactive | 842.0 (24.8%) | 253.0 (29.8%) | 219.0 (25.8%) | 199.0 (23.3%) | 171.0 (20.3%) |  |
| Median | 1,438.0 (42.4%) | 343.0 (40.4%) | 367.0 (43.2%) | 349.0 (40.9%) | 379.0 (45.1%) |  |
| Active | 1,113.0 (32.8%) | 253.0 (29.8%) | 264.0 (31.1%) | 305.0 (35.8%) | 291.0 (34.6%) |  |
| **HEI score** | 64.15 (0.45) | 64.13 (0.69) | 65.18 (0.65) | 65.46 (0.80) | 62.16 (0.88) | 0.018 |
| **Smoking status** |  |  |  |  |  | <0.001 |
| Current smoker | 669.0 (19.7%) | 106.0 (12.5%) | 134.0 (15.8%) | 200.0 (23.5%) | 229.0 (27.2%) |  |
| Ex-smoker | 1,005.0 (29.6%) | 185.0 (21.8%) | 221.0 (26.0%) | 284.0 (33.3%) | 315.0 (37.5%) |  |
| Never smoker | 1,718.0 (50.6%) | 558.0 (65.7%) | 495.0 (58.2%) | 368.0 (43.2%) | 297.0 (35.3%) |  |
| **BMI** | 30.06 (0.28) | 29.83 (0.86) | 29.87 (0.41) | 30.33 (0.33) | 30.13 (0.30) | 0.182 |
| **WC** | 101.02 (0.66) | 96.22 (1.75) | 98.32 (1.05) | 103.40 (0.82) | 104.20 (0.70) | <0.001 |
| **Hypertension** |  |  |  |  |  | 0.524 |
| Yes | 1,487.0 (43.8%) | 349.0 (41.1%) | 390.0 (45.9%) | 407.0 (47.7%) | 341.0 (40.5%) |  |
| No | 1,906.0 (56.2%) | 500.0 (58.9%) | 460.0 (54.1%) | 446.0 (52.3%) | 500.0 (59.5%) |  |
| **Diabetes** |  |  |  |  |  | 0.270 |
| Yes | 910.0 (26.8%) | 209.0 (24.6%) | 239.0 (28.1%) | 261.0 (30.6%) | 201.0 (23.9%) |  |
| No | 2,483.0 (73.2%) | 640.0 (75.4%) | 611.0 (71.9%) | 592.0 (69.4%) | 640.0 (76.1%) |  |
| **History of Malignancy** |  |  |  |  |  | 0.252 |
| Yes | 196.0 (5.8%) | 51.0 (6.0%) | 56.0 (6.6%) | 44.0 (5.2%) | 45.0 (5.4%) |  |
| No | 3,197.0 (94.2%) | 798.0 (94.0%) | 794.0 (93.4%) | 809.0 (94.8%) | 796.0 (94.6%) |  |
| **FIB4 index** | 0.95 (0.01) | 0.89 (0.03) | 1.00 (0.03) | 1.01 (0.03) | 0.89 (0.02) | 0.011 |
| **CRP, mg/dL** | 0.47 (0.02) | 0.58 (0.04) | 0.55 (0.03) | 0.46 (0.03) | 0.34 (0.01) | <0.001 |
| **HOMA-IR** | 4.03 (0.16) | 3.08 (0.18) | 3.81 (0.23) | 4.72 (0.36) | 4.16 (0.21) | <0.001 |
| ^†^ Wilcoxon rank-sum test for complex survey samples; chi-squared test with Rao & Scott's second-order correction.  TIBC, Total iron binding capacity; MASLD, Metabolic dysfunction-associated steatotic liver disease; HEI, Healthy eating index; BMI, body mass index; WC, waist circumference; CRP, C-reactive protein; HOMA-IR, Homeostatic Model Assessment for Insulin Resistance  Data are presented as mean (standard error) or number (percentage). | | | | | | |

| **Table S6.** Baseline characteristics of MASLD population by the mean corpuscular hemoglobin | | | | | | |
| --- | --- | --- | --- | --- | --- | --- |
| **Characteristic** | **Overall** | **Quartile 1**, <28.9 pg | **Quartile 2**, 28.9-30.0 pg | **Quartile 3**, 30.0-31.1 pg | **Quartile 4**, >31.1 pg | ***p*-value**^†^ |
| **Age (years)** | 46.90 (0.41) | 44.53 (0.81) | 46.95 (0.70) | 46.50 (0.72) | 48.87 (0.75) | 0.002 |
| **Sex** |  |  |  |  |  | 0.018 |
| Female | 1,864.0 (54.9%) | 560.0 (63.9%) | 449.0 (53.9%) | 462.0 (53.9%) | 393.0 (47.5%) |  |
| Male | 1,529.0 (45.1%) | 316.0 (36.1%) | 384.0 (46.1%) | 395.0 (46.1%) | 434.0 (52.5%) |  |
| **Ethnicity** |  |  |  |  |  | <0.001 |
| Mexican-American | 1,255.0 (37.0%) | 281.0 (32.1%) | 326.0 (39.1%) | 335.0 (39.1%) | 313.0 (37.8%) |  |
| Non-Hispanic white | 1,240.0 (36.5%) | 187.0 (21.3%) | 308.0 (37.0%) | 358.0 (41.8%) | 387.0 (46.8%) |  |
| Non-Hispanic black | 770.0 (22.7%) | 361.0 (41.2%) | 172.0 (20.6%) | 136.0 (15.9%) | 101.0 (12.2%) |  |
| Other | 128.0 (3.8%) | 47.0 (5.4%) | 27.0 (3.2%) | 28.0 (3.3%) | 26.0 (3.1%) |  |
| **Marry** |  |  |  |  |  | 0.130 |
| Yes | 2,217.0 (65.5%) | 511.0 (58.4%) | 561.0 (67.5%) | 574.0 (67.1%) | 571.0 (69.5%) |  |
| No | 1,166.0 (34.5%) | 364.0 (41.6%) | 270.0 (32.5%) | 281.0 (32.9%) | 251.0 (30.5%) |  |
| **PIR** |  |  |  |  |  | <0.001 |
| <1 | 769.0 (24.9%) | 251.0 (31.8%) | 184.0 (24.2%) | 179.0 (22.9%) | 155.0 (20.6%) |  |
| 1-5 | 2,046.0 (66.3%) | 496.0 (62.8%) | 511.0 (67.1%) | 531.0 (67.8%) | 508.0 (67.5%) |  |
| >5 | 272.0 (8.8%) | 43.0 (5.4%) | 66.0 (8.7%) | 73.0 (9.3%) | 90.0 (12.0%) |  |
| **Education** |  |  |  |  |  | 0.273 |
| <undergraduate | 2,737.0 (81.0%) | 724.0 (83.1%) | 675.0 (81.4%) | 679.0 (79.6%) | 659.0 (80.0%) |  |
| ≥undergraduate | 640.0 (19.0%) | 147.0 (16.9%) | 154.0 (18.6%) | 174.0 (20.4%) | 165.0 (20.0%) |  |
| **Physical activity** |  |  |  |  |  | 0.054 |
| Inactive | 842.0 (24.8%) | 250.0 (28.5%) | 208.0 (25.0%) | 196.0 (22.9%) | 188.0 (22.7%) |  |
| Median | 1,438.0 (42.4%) | 363.0 (41.4%) | 329.0 (39.5%) | 377.0 (44.0%) | 369.0 (44.6%) |  |
| Active | 1,113.0 (32.8%) | 263.0 (30.0%) | 296.0 (35.5%) | 284.0 (33.1%) | 270.0 (32.6%) |  |
| **HEI score** | 64.15 (0.45) | 62.98 (0.67) | 64.35 (0.92) | 65.64 (0.81) | 63.36 (0.66) | 0.073 |
| **Smoking status** |  |  |  |  |  | <0.001 |
| Current smoker | 669.0 (19.7%) | 126.0 (14.4%) | 147.0 (17.7%) | 170.0 (19.8%) | 226.0 (27.3%) |  |
| Ex-smoker | 1,005.0 (29.6%) | 219.0 (25.0%) | 256.0 (30.8%) | 281.0 (32.8%) | 249.0 (30.1%) |  |
| Never smoker | 1,718.0 (50.6%) | 531.0 (60.6%) | 429.0 (51.6%) | 406.0 (47.4%) | 352.0 (42.6%) |  |
| **BMI** | 30.06 (0.28) | 32.68 (0.48) | 29.86 (0.58) | 29.33 (0.28) | 29.17 (0.32) | <0.001 |
| **WC** | 101.02 (0.66) | 105.19 (0.97) | 100.76 (1.33) | 99.43 (0.71) | 99.97 (0.81) | <0.001 |
| **Hypertension** |  |  |  |  |  | 0.008 |
| Yes | 1,487.0 (43.8%) | 426.0 (48.6%) | 371.0 (44.5%) | 346.0 (40.4%) | 344.0 (41.6%) |  |
| No | 1,906.0 (56.2%) | 450.0 (51.4%) | 462.0 (55.5%) | 511.0 (59.6%) | 483.0 (58.4%) |  |
| **Diabetes** |  |  |  |  |  | 0.468 |
| Yes | 910.0 (26.8%) | 264.0 (30.1%) | 224.0 (26.9%) | 221.0 (25.8%) | 201.0 (24.3%) |  |
| No | 2,483.0 (73.2%) | 612.0 (69.9%) | 609.0 (73.1%) | 636.0 (74.2%) | 626.0 (75.7%) |  |
| **History of Malignancy** |  |  |  |  |  | 0.325 |
| Yes | 196.0 (5.8%) | 35.0 (4.0%) | 46.0 (5.5%) | 64.0 (7.5%) | 51.0 (6.2%) |  |
| No | 3,197.0 (94.2%) | 841.0 (96.0%) | 787.0 (94.5%) | 793.0 (92.5%) | 776.0 (93.8%) |  |
| **FIB4 index** | 0.95 (0.01) | 0.84 (0.03) | 0.93 (0.02) | 0.93 (0.03) | 1.04 (0.03) | <0.001 |
| **CRP, mg/dL** | 0.47 (0.02) | 0.66 (0.05) | 0.44 (0.03) | 0.41 (0.02) | 0.42 (0.02) | <0.001 |
| **HOMA-IR** | 4.03 (0.16) | 4.81 (0.36) | 3.71 (0.25) | 3.86 (0.29) | 3.92 (0.25) | <0.001 |
| ^†^ Wilcoxon rank-sum test for complex survey samples; chi-squared test with Rao & Scott's second-order correction.  TIBC, Total iron binding capacity; MASLD, Metabolic dysfunction-associated steatotic liver disease; HEI, Healthy eating index; BMI, body mass index; WC, waist circumference; CRP, C-reactive protein; HOMA-IR, Homeostatic Model Assessment for Insulin Resistance  Data are presented as mean (standard error) or number (percentage). | | | | | | |

| **Table S7.** Baseline characteristics of MASLD population by the mean corpuscular volume | | | | | | |
| --- | --- | --- | --- | --- | --- | --- |
| **Characteristic** | **Overall** | **Quartile 1**, <85.9 fL | **Quartile 2**, 85.9-89.0 fL | **Quartile 3**, 89.0-91.8 fL | **Quartile 4**, >91.8 fL | ***p*-value**^†^ |
| **Age (years)** | 46.90 (0.41) | 44.08 (0.81) | 45.80 (0.80) | 47.33 (0.70) | 49.69 (0.58) | <0.001 |
| **Sex** |  |  |  |  |  | 0.986 |
| Female | 1,864.0 (54.9%) | 508.0 (59.6%) | 477.0 (56.1%) | 454.0 (53.5%) | 425.0 (50.5%) |  |
| Male | 1,529.0 (45.1%) | 344.0 (40.4%) | 374.0 (43.9%) | 395.0 (46.5%) | 416.0 (49.5%) |  |
| **Ethnicity** |  |  |  |  |  | <0.001 |
| Mexican-American | 1,255.0 (37.0%) | 286.0 (33.6%) | 344.0 (40.4%) | 320.0 (37.7%) | 305.0 (36.3%) |  |
| Non-Hispanic white | 1,240.0 (36.5%) | 200.0 (23.5%) | 314.0 (36.9%) | 351.0 (41.3%) | 375.0 (44.6%) |  |
| Non-Hispanic black | 770.0 (22.7%) | 322.0 (37.8%) | 165.0 (19.4%) | 145.0 (17.1%) | 138.0 (16.4%) |  |
| Other | 128.0 (3.8%) | 44.0 (5.2%) | 28.0 (3.3%) | 33.0 (3.9%) | 23.0 (2.7%) |  |
| **Marry** |  |  |  |  |  | 0.283 |
| Yes | 2,217.0 (65.5%) | 512.0 (60.1%) | 571.0 (67.3%) | 557.0 (65.8%) | 577.0 (69.1%) |  |
| No | 1,166.0 (34.5%) | 340.0 (39.9%) | 278.0 (32.7%) | 290.0 (34.2%) | 258.0 (30.9%) |  |
| **PIR** |  |  |  |  |  | 0.009 |
| <1 | 769.0 (24.9%) | 238.0 (31.1%) | 200.0 (25.5%) | 158.0 (20.7%) | 173.0 (22.4%) |  |
| 1-5 | 2,046.0 (66.3%) | 484.0 (63.2%) | 520.0 (66.4%) | 525.0 (68.7%) | 517.0 (66.8%) |  |
| >5 | 272.0 (8.8%) | 44.0 (5.7%) | 63.0 (8.0%) | 81.0 (10.6%) | 84.0 (10.9%) |  |
| **Education** |  |  |  |  |  | 0.454 |
| <undergraduate | 2,737.0 (81.0%) | 706.0 (83.1%) | 677.0 (80.3%) | 671.0 (79.2%) | 683.0 (81.6%) |  |
| ≥undergraduate | 640.0 (19.0%) | 144.0 (16.9%) | 166.0 (19.7%) | 176.0 (20.8%) | 154.0 (18.4%) |  |
| **Physical activity** |  |  |  |  |  | 0.766 |
| Inactive | 842.0 (24.8%) | 232.0 (27.2%) | 218.0 (25.6%) | 192.0 (22.6%) | 200.0 (23.8%) |  |
| Median | 1,438.0 (42.4%) | 363.0 (42.6%) | 343.0 (40.3%) | 375.0 (44.2%) | 357.0 (42.4%) |  |
| Active | 1,113.0 (32.8%) | 257.0 (30.2%) | 290.0 (34.1%) | 282.0 (33.2%) | 284.0 (33.8%) |  |
| **HEI score** | 64.15 (0.45) | 63.36 (0.74) | 64.21 (0.80) | 64.97 (0.72) | 63.91 (0.77) | 0.338 |
| **Smoking status** |  |  |  |  |  | 0.001 |
| Current smoker | 669.0 (19.7%) | 121.0 (14.2%) | 147.0 (17.3%) | 166.0 (19.6%) | 235.0 (27.9%) |  |
| Ex-smoker | 1,005.0 (29.6%) | 224.0 (26.3%) | 254.0 (29.9%) | 278.0 (32.7%) | 249.0 (29.6%) |  |
| Never smoker | 1,718.0 (50.6%) | 507.0 (59.5%) | 449.0 (52.8%) | 405.0 (47.7%) | 357.0 (42.4%) |  |
| **BMI** | 30.06 (0.28) | 32.49 (0.42) | 30.23 (0.51) | 29.25 (0.28) | 28.84 (0.31) | <0.001 |
| **WC** | 101.02 (0.66) | 105.64 (0.96) | 101.23 (1.15) | 99.45 (0.69) | 98.81 (0.90) | <0.001 |
| **Hypertension** |  |  |  |  |  | 0.031 |
| Yes | 1,487.0 (43.8%) | 412.0 (48.4%) | 366.0 (43.0%) | 362.0 (42.6%) | 347.0 (41.3%) |  |
| No | 1,906.0 (56.2%) | 440.0 (51.6%) | 485.0 (57.0%) | 487.0 (57.4%) | 494.0 (58.7%) |  |
| **Diabetes** |  |  |  |  |  | 0.566 |
| Yes | 910.0 (26.8%) | 245.0 (28.8%) | 240.0 (28.2%) | 225.0 (26.5%) | 200.0 (23.8%) |  |
| No | 2,483.0 (73.2%) | 607.0 (71.2%) | 611.0 (71.8%) | 624.0 (73.5%) | 641.0 (76.2%) |  |
| **History of Malignancy** |  |  |  |  |  | 0.219 |
| Yes | 196.0 (5.8%) | 33.0 (3.9%) | 46.0 (5.4%) | 61.0 (7.2%) | 56.0 (6.7%) |  |
| No | 3,197.0 (94.2%) | 819.0 (96.1%) | 805.0 (94.6%) | 788.0 (92.8%) | 785.0 (93.3%) |  |
| **FIB4 index** | 0.95 (0.01) | 0.84 (0.03) | 0.90 (0.02) | 0.94 (0.03) | 1.09 (0.03) | <0.001 |
| **CRP, mg/dL** | 0.47 (0.02) | 0.63 (0.05) | 0.45 (0.02) | 0.41 (0.02) | 0.42 (0.02) | <0.001 |
| **HOMA-IR** | 4.03 (0.16) | 4.65 (0.29) | 4.16 (0.32) | 3.64 (0.20) | 3.76 (0.27) | <0.001 |
| ^†^ Wilcoxon rank-sum test for complex survey samples; chi-squared test with Rao & Scott's second-order correction.  TIBC, Total iron binding capacity; MASLD, Metabolic dysfunction-associated steatotic liver disease; HEI, Healthy eating index; BMI, body mass index; WC, waist circumference; CRP, C-reactive protein; HOMA-IR, Homeostatic Model Assessment for Insulin Resistance  Data are presented as mean (standard error) or number (percentage). | | | | | | |

| **Table S8.** Baseline characteristics of MASLD population by the mean corpuscular hemoglobin concentration | | | | | | |
| --- | --- | --- | --- | --- | --- | --- |
| **Characteristic** | **Overall** | **Quartile 1**, <331.5 g/L | **Quartile 2**, 331.5-337.0 g/L | **Quartile 3**, 337.0-342.0 g/L | **Quartile 4**, >342.0 g/L | ***p*-value**^†^ |
| **Age (years)** | 46.90 (0.41) | 48.22 (0.90) | 47.31 (0.71) | 47.06 (0.60) | 45.46 (1.15) | 0.347 |
| **Sex** |  |  |  |  |  | <0.001 |
| Female | 1,864.0 (54.9%) | 578.0 (65.5%) | 474.0 (55.0%) | 454.0 (52.9%) | 358.0 (45.3%) |  |
| Male | 1,529.0 (45.1%) | 304.0 (34.5%) | 388.0 (45.0%) | 405.0 (47.1%) | 432.0 (54.7%) |  |
| **Ethnicity** |  |  |  |  |  | <0.001 |
| Mexican-American | 1,255.0 (37.0%) | 273.0 (31.0%) | 308.0 (35.7%) | 323.0 (37.6%) | 351.0 (44.4%) |  |
| Non-Hispanic white | 1,240.0 (36.5%) | 206.0 (23.4%) | 322.0 (37.4%) | 357.0 (41.6%) | 355.0 (44.9%) |  |
| Non-Hispanic black | 770.0 (22.7%) | 364.0 (41.3%) | 199.0 (23.1%) | 146.0 (17.0%) | 61.0 (7.7%) |  |
| Other | 128.0 (3.8%) | 39.0 (4.4%) | 33.0 (3.8%) | 33.0 (3.8%) | 23.0 (2.9%) |  |
| **Marry** |  |  |  |  |  | 0.002 |
| Yes | 2,217.0 (65.5%) | 512.0 (58.2%) | 574.0 (66.9%) | 568.0 (66.2%) | 563.0 (71.5%) |  |
| No | 1,166.0 (34.5%) | 368.0 (41.8%) | 284.0 (33.1%) | 290.0 (33.8%) | 224.0 (28.5%) |  |
| **PIR** |  |  |  |  |  | <0.001 |
| <1 | 769.0 (24.9%) | 242.0 (30.3%) | 177.0 (22.5%) | 182.0 (23.2%) | 168.0 (23.3%) |  |
| 1-5 | 2,046.0 (66.3%) | 510.0 (63.9%) | 534.0 (68.0%) | 526.0 (67.1%) | 476.0 (66.1%) |  |
| >5 | 272.0 (8.8%) | 46.0 (5.8%) | 74.0 (9.4%) | 76.0 (9.7%) | 76.0 (10.6%) |  |
| **Education** |  |  |  |  |  | 0.124 |
| <undergraduate | 2,737.0 (81.0%) | 730.0 (83.1%) | 698.0 (81.4%) | 690.0 (80.6%) | 619.0 (78.8%) |  |
| ≥undergraduate | 640.0 (19.0%) | 148.0 (16.9%) | 159.0 (18.6%) | 166.0 (19.4%) | 167.0 (21.2%) |  |
| **Physical activity** |  |  |  |  |  | 0.007 |
| Inactive | 842.0 (24.8%) | 253.0 (28.7%) | 223.0 (25.9%) | 189.0 (22.0%) | 177.0 (22.4%) |  |
| Median | 1,438.0 (42.4%) | 341.0 (38.7%) | 373.0 (43.3%) | 388.0 (45.2%) | 336.0 (42.5%) |  |
| Active | 1,113.0 (32.8%) | 288.0 (32.7%) | 266.0 (30.9%) | 282.0 (32.8%) | 277.0 (35.1%) |  |
| **HEI score** | 64.15 (0.45) | 63.21 (0.94) | 64.07 (0.69) | 64.86 (0.86) | 64.12 (1.01) | 0.640 |
| **Smoking status** |  |  |  |  |  | 0.303 |
| Current smoker | 669.0 (19.7%) | 161.0 (18.3%) | 163.0 (18.9%) | 179.0 (20.9%) | 166.0 (21.0%) |  |
| Ex-smoker | 1,005.0 (29.6%) | 229.0 (26.0%) | 259.0 (30.0%) | 255.0 (29.7%) | 262.0 (33.2%) |  |
| Never smoker | 1,718.0 (50.6%) | 492.0 (55.8%) | 440.0 (51.0%) | 424.0 (49.4%) | 362.0 (45.8%) |  |
| **BMI** | 30.06 (0.28) | 30.16 (0.43) | 30.04 (0.43) | 30.04 (0.46) | 30.04 (0.32) | 0.898 |
| **WC** | 101.02 (0.66) | 99.66 (1.16) | 100.77 (1.04) | 101.33 (0.98) | 101.84 (0.85) | 0.220 |
| **Hypertension** |  |  |  |  |  | 0.634 |
| Yes | 1,487.0 (43.8%) | 417.0 (47.3%) | 368.0 (42.7%) | 362.0 (42.1%) | 340.0 (43.0%) |  |
| No | 1,906.0 (56.2%) | 465.0 (52.7%) | 494.0 (57.3%) | 497.0 (57.9%) | 450.0 (57.0%) |  |
| **Diabetes** |  |  |  |  |  | 0.724 |
| Yes | 910.0 (26.8%) | 239.0 (27.1%) | 231.0 (26.8%) | 222.0 (25.8%) | 218.0 (27.6%) |  |
| No | 2,483.0 (73.2%) | 643.0 (72.9%) | 631.0 (73.2%) | 637.0 (74.2%) | 572.0 (72.4%) |  |
| **History of Malignancy** |  |  |  |  |  | 0.139 |
| Yes | 196.0 (5.8%) | 49.0 (5.6%) | 58.0 (6.7%) | 48.0 (5.6%) | 41.0 (5.2%) |  |
| No | 3,197.0 (94.2%) | 833.0 (94.4%) | 804.0 (93.3%) | 811.0 (94.4%) | 749.0 (94.8%) |  |
| **FIB4 index** | 0.95 (0.01) | 0.98 (0.03) | 0.98 (0.04) | 0.93 (0.02) | 0.93 (0.03) | 0.654 |
| **CRP, mg/dL** | 0.47 (0.02) | 0.51 (0.03) | 0.49 (0.04) | 0.43 (0.02) | 0.45 (0.03) | 0.825 |
| **HOMA-IR** | 4.03 (0.16) | 3.47 (0.24) | 4.02 (0.31) | 4.34 (0.32) | 4.08 (0.22) | 0.225 |
| ^†^ Wilcoxon rank-sum test for complex survey samples; chi-squared test with Rao & Scott's second-order correction.  TIBC, Total iron binding capacity; MASLD, Metabolic dysfunction-associated steatotic liver disease; HEI, Healthy eating index; BMI, body mass index; WC, waist circumference; CRP, C-reactive protein; HOMA-IR, Homeostatic Model Assessment for Insulin Resistance  Data are presented as mean (standard error) or number (percentage). | | | | | | |
